# Age, morbidity, or something else? A residual approach using microdata to measure the impact of technological progress on health care expenditure

**DOI:** 10.1002/hec.4500

**Published:** 2022-03-31

**Authors:** Mauro Laudicella, Paolo Li Donni, Kim Rose Olsen, Dorte Gyrd‐Hansen

**Affiliations:** ^1^ Danish Centre for Health Economics ‐ DaCHE University of Southern Denmark Odense Denmark; ^2^ Economics Department University of Palermo Palermo Italy

**Keywords:** aging, health care expenditure, morbidity, technological, time‐to‐death

## Abstract

This study measures the increment of health care expenditure (HCE) that can be attributed to technological progress and change in medical practice by using a residual approach and microdata. We examine repeated cross‐sections of individuals experiencing an initial health shock at different point in time over a 10‐year window and capture the impact of unobservable technology and medical practice to which they are exposed after allowing for differences in health and socioeconomic characteristics. We decompose the residual increment in the part that is due to the effect of delaying time to death, that is, individuals surviving longer after a health shock and thus contributing longer to the demand of care, and the part that is due to increasing intensity of resource use, that is, the basket of services becoming more expensive to allow for the cost of innovation. We use data from the Danish National Health System that offers universal coverage and is free of charge at the point of access. We find that technological progress and change in medical practice can explain about 60% of the increment of HCE, in line with macroeconomic studies that traditionally investigate this subject.

## 1 INTRODUCTION

High‐income countries are experiencing a rapid growth of health care expenditure (HCE) that seems to outpace demographic growth and aging of their population jeopardizing the fiscal sustainability of their health systems (OECD, 2006, 2015). The first objective of this study is to measure the effect of non‐demographic and non‐health related drivers, such as technological progress and change in medical practice, on the increment of HCE over time. In spite of the relevance of these factors frequently being mentioned in the literature, research measuring their impact on HCE is sparse due to a lack of agreement on their definition and conceptualization of appropriate indicators (Chernew & Newhouse, 2011; Martín et al., 2011). The approach proposed in this paper is to measure their effect as a residual increment (RI) of HCE after controlling for observable demographic and health drivers using microdata. We performed a repeated cross‐section analysis on individuals experiencing an initial health shock to capture the impact of unobservable technology and medical practice to which they are exposed when they experience the shock, allowing for the data generating process that links variation in age and morbidity to variation in HCE over time. The identification of the effect of technological progress and change in medical practice on HCE is achieved from the assignment of individuals to their initial health shock over a time window of 10 years, which occurs with some degree of randomness with respect to the technology available to treat them after the shock, and a large battery of individual indicators adjusting for variation in health and sociodemographic characteristics. We find evidence that the RI account for 60% of the total increment of HCE in our study population.

The second objective of this study is to decompose the RI of HCE in the part that is due to delaying time to death (TTD), that is, individuals surviving longer after a health shock, and the part that is due to increasing intensity of resource use, that is, individuals consuming more resources per unit of time. New technologies and medical practices may generate an increase in HCE over time through two different channels: first, the basket of services accessed by patients becomes more expensive to allow for the cost of innovation; second, patients are able to delay the TTD associated with their health condition and thus they can potentially contribute to the demand for health care for longer. Numerous studies demonstrate that approaching TTD prompts an exponential increment of HCE leading to the suggestion that HCE might reduce in the future as individual experience increasing life expectancy over time and hence delay their TTD (Felder et al., 2010; Werblow et al., 2007; Wong et al., 2011; Zweifel et al., 1999). However, this hypothesis has not been formally tested and it is likely to depend on the source of the increment in life expectancy. HCE is likely to fall if the increment in life expectancy is due to slowing down the process of aging gaining additional life years free from morbidity and disability, as suggested by the compression of morbidities hypothesis (Fries et al., 2011; Geyer et al., 2018; Manton, 2008). In contrast, HCE might increase if additional life years attracts morbidities and disabilities. Such a scenario might arise when additional life years are gained through new medical technologies and practices that are capable of saving the life of patients, but unable to grant them a full recovery from disease leaving them permanently frailer after the intervention (Gruenberg, 2005; Laudicella et al., 2013; Laudicella, Martin, et al., 2018). In this study, we provide evidence on the impact of delaying TTD that stems from the latter source. We find that about one fourth of the RI of HCE can be attributed to delaying TTD, while the rest is due to increasing intensity of resource use. However, the impact of delaying TTD is heterogeneous according to the health conditions that prompt the health shock.

Econometric analysis is based on a three‐part estimator predicting the probability of surviving, the probability of using health care services, and the conditional HCE over the time elapsed after the health shock. This estimator was originally developed by Basu and Manning (2010) for the analysis of episodes‐of‐illness costs over time. We use the BM‐estimator to model the impact of a health shock on HCE over two dimensions of time describing our data generating process: calendar time when individuals experience the shock and elapsed time after the shock.

The study is based on a rich dataset covering the whole population of residents in Denmark age 50+ using inpatient and outpatient hospital services. The dataset includes very accurate information on individual's morbidity and DRG tariffs that are used to reimburse hospital services. We study the Danish National Health System (DNHS) that is free of charge at the point of use and offers a universal coverage to its population. The DNHS offers an ideal setting to assess variation in HCE over time as the use of health services is not confounded by variation in ability to pay or access to health insurance.

### 1.1 Literature background

Numerous studies suggest TTD and morbidity are key drivers of HCE, whereas aging captures the effect of these factors when they are omitted from the analysis; hence age has been labeled a red herring (Felder et al., 2010; Werblow et al., 2007; Wong et al., 2011; Zweifel et al., 1999). Research based on hospital administrative data reinforces the case for morbidity as one the main drivers of HCE suggesting that TTD captures the effect of unmeasured morbidity, and thus the relationship between TTD and HCE could be another red herring (de Meijer et al., 2011; Howdon & Rice, 2018; Moore et al., 2017; Shang & Goldman, 2008). However, the literature is still debating the extent to which age and morbidity are the causes of the increment of HCE observed in the past, and whether they are good predictors of its growth in the future (Breyer & Lorenz, 2020; de Meijer et al., 2013; Dormont et al., 2006). Epidemiological studies suggest that morbidity follows a compression process characterized by increasing life expectancy and non‐increasing total number of years lived with disability (Geyer et al., 2018; Manton, 2008; Payne et al., 2007). Also, evidence on TTD suggests that increasing life expectancy of the population could contribute to reducing HCE over time as individuals postpone the increase in HCE associated with end of life (Breyer et al., 2015; de Meijer et al., 2011; Howdon & Rice, 2018; van Baal & Wong, 2012).

Despite the large body of research on the response of HCE to demographic and health related drivers, little attention has been paid to the variation that remains unexplained after controlling for these factors. Some studies move in this direction by decomposing the increment of HCE over time into the part that is due to variation in the distribution of its drivers and the part that is due to variation in their effects following Oxaca type and Chernozhukov type decomposition approaches (de Meijer et al., 2013; Dormont et al., 2006; Rice & Aragon, 2018). Variation in the distribution of the drivers is then attributed to changes in demographic and health factors in the population, while variation in their effect is attributed to technological progress and changes in medical practice.

Finally, a residual approach to measure the impact of technological progress on HCE growth has been adopted in many macroeconomic studies (Finkelstein, 2007; Newhouse, 1992; Peden & Freeland, 1998; Smith et al., 2009). However, to the extent of our knowledge, this is the first application using microdata that allows for an accurate control over observable drivers, such as morbidity, and avoids assumptions on factors encouraging technological progress, such as income elasticity and insurance coverage, which often underpin macroeconomic studies (Chernew & Newhouse, 2011).

## 2 DATA

We used data extracted from the Danish National Patient Register including all elective and emergency admissions to hospital and outpatient visits occurring between 2000 and 2017. We had access to information on patient admission and discharge date, each admission and outpatient visit includes information on primary diagnosis and up to 20 secondary diagnosis reported using ICD‐10 codes. Every hospital discharge and outpatient visit attract a DRG tariff from which HCE is derived; DRG tariffs are reported in our data from 2003 onwards. All residents of Denmark are identified by a unique identification number that is used to follow them through the Danish National Patient Register and to link them to a number of other registers at the individual level. We linked data on date of death from the Register of Causes of Death, individual annual income from the Income Statistics Register, and living alone status from the Central Person Register (see Thygesen et al. (2011) for an overview on Danish registries).

### 2.1 Institutional framework

The DNHS offers a universal coverage to residents in Denmark and free access to primary and secondary care services, which are funded by the taxpayers. Secondary care, including elective and emergency outpatient and inpatient services, is delivered by a network of 21 large multi‐service hospitals, which are non‐profit public organizations serving a local population of 250,000 residents and managed by the 5 Danish Regions. Access to elective care is managed by General Practitioners (GPs), who are the gate keepers of the system, while emergency care can be accessed by calling an emergency number managed by the Emergency Medical Coordination Centers, which assess the urgency of the call and direct the patients to the closest Emergency Department available. Hospital services are reimbursed by the Danish Regions through a system of DRG tariffs centrally determined by the Department of Health on the basis of the average costs reported annually by hospitals. DRGs were initially introduced in 2000 to facilitate payments for patients choosing to receive their treatment in a different administrative area than their area of residence; 3 years later they were extended to all patients as a tool to incentivize hospital productivity and in 2005 were officially adopted as a reimbursement system. The Hospital sector underwent a reorganization in 2007 aiming at reducing costs and improving the quality of services; acute hospitals were reorganized into 21 large multiservice organizations with highly specialized treatments, such as surgery for lung cancer, heart surgery, transplants or treatment of serious burns, centralized in 1–3 locations in the country (Christiansen & Vrangbaek, 2018). This reform undoubtedly contributed to the trajectory of HCE and can be considered as one of the forces that contributed to the introduction of new technology and medical practice in Denmark.

### 2.2 Study population

Our study population includes individuals age 50+ exposed to an initial health shock in different calendar years over a 10‐year window,^1^ from 2005 to 2014. A health shock is defined as an emergency admission for any cause with a length of stay of at least 1 day.^2^, ^3^ Individuals enter the study at the time of their initial health shock, that is, the first shock in a moving time window of 5 years, for example, an individual enters the study in 2005 if she experiences a health shock in 2005 and no shocks in 2000–2004. Then, we follow each individual 2 years before and 3 years after her initial health shock and extract information on her HCE, health and sociodemographic characteristics. Hence, our study population is constructed using data from 2000 to 2017 allowing for the initial health shock and the follow up period, although exposure to new technology and medical practice is assessed between 2005 and 2014. It is worth noting that our definition of initial health shock aims to capture a deterioration of individual's health that leads to an unplanned event, that is, an emergency admission, rather than a deterioration of health that leads to a new health condition. For instance, patients experiencing an emergency admission may have been diagnosed earlier and may already have been placed on a course of elective treatment. Such a definition allows us to capture a large population of 962,794 individuals who are potentially frequent users of the Health System. For instance, individuals experiencing an initial health shock in 2014 account for 23.5% of national HCE for all hospital inpatient and outpatient services in the same year and for 8.2% of all users in the same age group.

### 2.3 Dependent variable

Our dependent variable is the HCE for hospital inpatient and outpatient services accessed by individuals in our study population at 2017 price level. This includes HCE for pharmaceuticals delivered during inpatient admissions and outpatient visits. We calculate HCE over different time windows from the onset of the health shock to which individuals are exposed, that is, 365 days and 1095 days, and we indicate the specific time window adopted in the method section and in the results section to avoid confusion. HCE is measured by a system of DRG tariffs that the Danish Regions pay to hospitals on the basis of the services delivered to patients every year. The DRG payment system was officially introduced in 2005, although DRG tariffs were calculated and reported since 2003. The DRG tariffs are updated every year to allow for changes in the cost of services; updates are based on the national average cost of each service calculated from hospital cost returns. Official calculations for inflation are produced by the Danish Regions annually and separately for primary care, secondary care, and pharmaceuticals; building blocks of these calculations are: the report over the business cycle from the Ministry of Economic Affairs and the Interior Ministry's financial statement; wage developments determined by all collective agreements with labor unions; price developments determined by a basket of goods and services, which include fuel, food, transport, land and buildings, and service procurement. The official inflation coefficients reported for hospital services were used to adjust HCE at 2017 price level.^4^

### 2.4 Control variables

We use a large basket of individual indicators capturing health and sociodemographic characteristics to control for individual heterogeneity in the analysis. Health indicators were based on individual health conditions reported in the hospital records at the time of the health shock and in the 2 years before the shock, including: age, gender, indicator for primary diagnosis and total secondary diagnoses, Charlson index measuring mortality risk, comorbidity indicators for acute and chronic conditions affecting mortality risk (acute myocardial infarction [AMI], congestive heart failure, peripheral vascular disease, cerebrovascular disease, dementia, chronic obstructive pulmonary disease, rheumatoid disease, peptic ulcer, liver disease, diabetes, and renal disease, cancer). Indicators for the primary diagnosis include a total of 1005 distinct indicators based on the ICD‐10 system for classification of diseases reported at the time of the health shock. The Charlson index and comorbidity indicators are calculated by using hospital records at the time of the health shock and 2 years before the shock. Finally, we included indicators for individual HCE occurring 1 year and 2 years before the health shock to control for heterogeneity in utilization of care before the shock.

The socioeconomic indicators include individual income, living alone status, and migrant status. Individuals not living alone might receive informal care reducing utilization of other types of care (de Meijer et al., 2011).

### 2.5 Descriptive statistics

**FIGURE 1:**
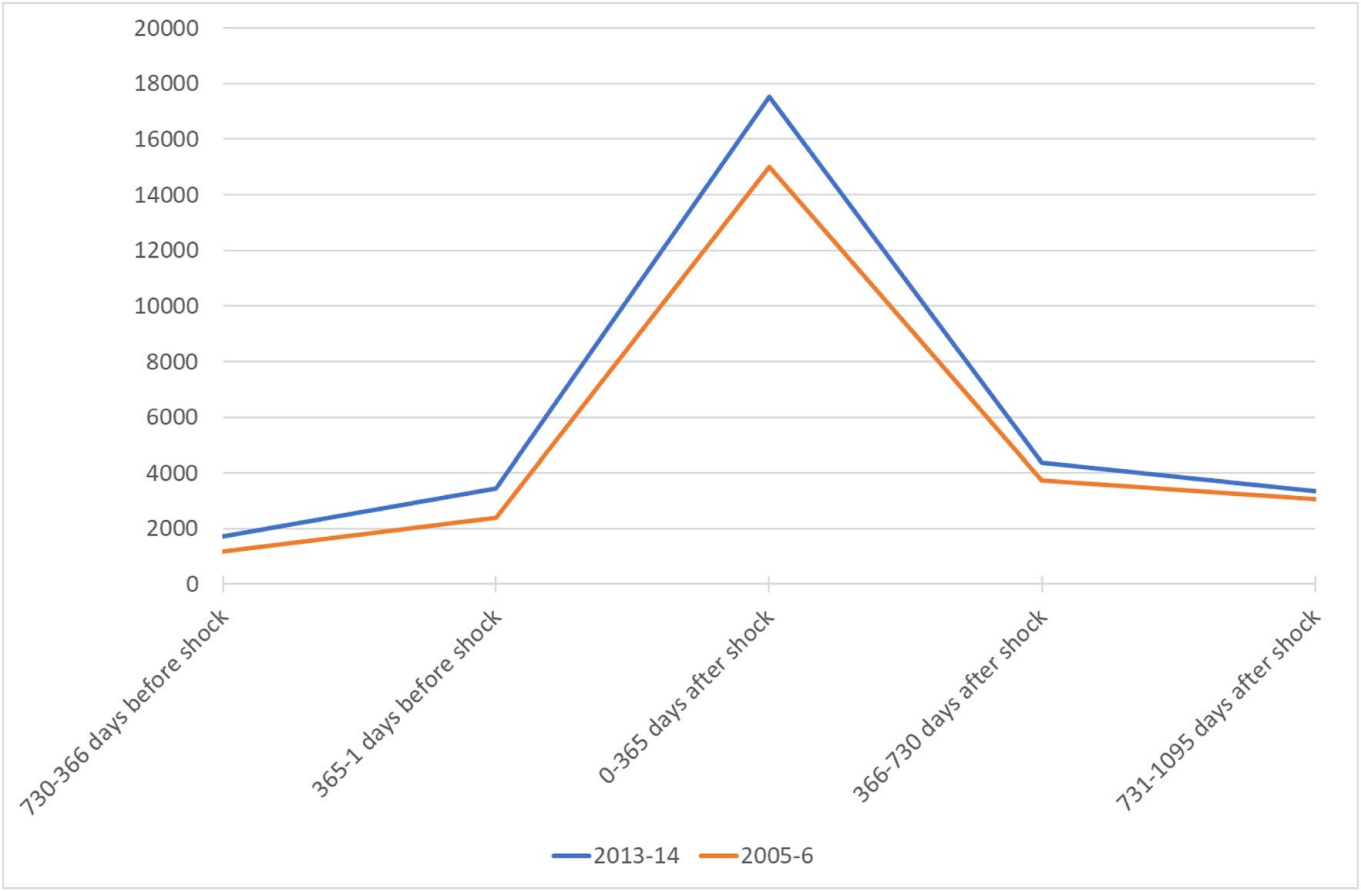
Health care expenditure after an initial health shock in 2005‐6 and 2013‐14. Prices reported in Euros at 2017 level

Figure 1 shows the distribution of HCE before and after an initial health shock occurring at the two end points of our study, that is, 2005‐6 and 2013‐14. HCE is reported in Euros at 2017 price level and observations are grouped in 2‐year intervals. Individuals experiencing a health shock in 2013‐14 consumed health resources for 17,541 € in the first year after the shock (i.e., the interval 0–365 days including the shock), and 4358 and 3341 € in the second and third year after the shock respectively (i.e., the intervals 366–730 days and 731–1095 days after the shock). In contrast, HCE in patients experiencing a health shock in 2005‐6, was considerably lower totaling 15,004 € in the first year after the shock and 3748 € and 3052 € in the second and third year.

Table 1 reports descriptive statistics for individuals experiencing a health shock at the two end points of our study period. Patients with a health shock in 2013‐14 survive 4.86 days longer and are 2.53% points less likely to face TTD than patients with a health shock in 2005‐6. However, the former are 0.41 years older, have 0.06 points higher Charlson index for mortality risk and have 0.09 more diagnoses than the latter. In terms of prevalence of specific morbidities, patients with a health shock in 2013‐14 are less likely to have: AMI (−0.93% points), congestive heart failure (−0.47 pp), cerebrovascular disease (i.e., strokes; −0.82 pp), and peptic ulcer (−0.68 pp); in contrast, they are more likely to have: chronic obstructive pulmonary disease (0.39 pp), diabetes without complications (0.79 pp), renal disease (0.56 pp), cancer (2.08 pp) and metastatic cancer (0.30 pp). With respect to socioeconomic status they are less likely to live alone (−0.88 pp), more likely to be a migrant (1.33 pp), and their annual income is 2607 € higher in real terms than individuals having a health shock 8 years before. Finally, Table 1 shows that the total number of individuals experiencing a health shock increased by 3.28% moving from 188,275 to 194,459 in the 8‐year period examined.

**TABLE 1:** Difference in the characteristics of individuals with a health shock in 2014‐13 and 2005‐6

	Health shock 2013‐14			Heath shock 2005‐6			Difference
	Patients	Mean	s.d.	Patients	Mean	s.d.	
Health Care Expenditure (Euros)							
0–365 days after shock	194,459	17541.01	25462.22	188,275	15004.84	19808.74	2536.17
366–730 days after shock	194,459	4358.17	11914.09	188,275	3748.27	10359.22	609.90
731–1095 days after shock	194,459	3341.87	9907.74	188,275	3052.57	8919.10	289.30
365‐1 day before shock	194,459	3456.06	9068.51	188,275	2401.08	7374.29	1054.99
730‐366 days before shock	194,459	1718.42	5488.90	188,275	1168.62	4141.11	549.80
Time to Death							
TTD (days from shock to death)	42,121	353.403	334.8776	45,547	348.5398	335.7149	4.8632
Entering TTD (within 3 years from shock)	194,459	21.66%		188,275	24.19%		−2.53%
Demographic characteristics							
Female	194,459	50.56%		188,275	51.90%		−1.34%
Age	194,459	69.04	11.22	188,275	68.63	11.4322	0.41
Comorbidities at the time of the shock and up to 730 days before							
Total diagnoses (at the time of the shock)	194,459	2.0385	1.3995	188,275	1.945	1.2338	0.0935
Charlson index	194,459	0.8578	1.4515	188,275	0.8005	1.3465	0.0573
AMI	194,459	4.60%		188,275	5.53%		−0.93%
Congestive heart failure	194,459	3.73%		188,275	4.20%		−0.47%
Peripheral vascular disease	194,459	2.99%		188,275	2.76%		0.23%
Cerebrovascular disease	194,459	9.24%		188,275	10.06%		−0.82%
Dementia	194,459	1.96%		188,275	2.12%		−0.16%
Chronic obstructive pulmonary dis.	194,459	6.80%		188,275	6.41%		0.39%
Rheumatoid disease	194,459	1.97%		188,275	1.86%		0.11%
Peptic ulcer	194,459	1.36%		188,275	2.04%		−0.68%
Liver disease (mild)	194,459	0.87%		188,275	0.83%		0.04%
Liver disease (severe)	194,459	0.35%		188,275	0.31%		0.04%
Diabetes	194,459	7.24%		188,275	6.45%		0.79%
Diabetes complications	194,459	1.68%		188,275	1.67%		0.01%
Renal disease	194,459	1.65%		188,275	1.09%		0.56%
Cancer	194,459	11.95%		188,275	9.87%		2.08%
Metastatic Cancer	194,459	2.43%		188,275	2.13%		0.30%
Socioeconomic characteristics							
Living alone	194,459	41.65%		188,275	42.53%		−0.88%
Migrant	194,459	5.51%		188,275	4.18%		1.33%
Income (x1,000 €)	194,459	29.2736	38.3464	188,275	26.6662	38.4559	2.6074
Length of stay (at the time of the shock)	194,459	3.72	5.34	188,275	5.06	8.05	−1.34

Abbreviation: AMI, acute myocardial infarction.

## 3 METHODS

The empirical analysis consists of two parts. In the first part, we measure the RI of HCE in each calendar year by using a repeated cross‐section analysis. In the second part, we decompose the RI of HCE into delaying TTD effect and intensity effect by applying a time‐to‐event longitudinal analysis.

### 3.1 Measuring the RI of HCE

We model HCE by using a generalised linear model (GLM) with gamma distribution and log link function. Box‐Cox tests and Modified Park tests were used to select the appropriate link function and family function:

(1) $$E\left(HCE_{i}|x_{i}\right)=\exp\left(t+\beta X_{i}\right)$$

$HCE_{i}$ measures the total amount paid for inpatient and outpatient services accessed by individual *“i”* from the onset of the health shock to 365 days after. The RI of HCE is captured by a vector of dummies, *t*, for the calendar year of the initial health shock after controlling for observable individual health and sociodemographic characteristics in $X_{i}$. Our identification strategy is to use variation in HCE generated by individuals with similar observables health and sociodemographic characteristics exposed to different unobservable technology and medical practice in different calendar years. This approach aims to address the data generating process that links age and morbidity and unobservable drivers, such as technology and medical practice, to HCE over time in line with the objectives of our study. Typically, individuals experience a specific age and health shock only in one calendar time point during their lifetime, since within individual variation in these factors goes only in one direction over time. Therefore, it would be difficult to disentangle the effect of variation in observable and unobservable drivers of HCE over time by following the same individual over calendar time, for example, by using a panel data approach.^5^ Finally, including individuals at the time of their initial health shock allows us to mitigate the confounding effect of technology and medical practice to which they were exposed in the past, which could influence their probability of being included in the study (e.g., surviving a previous health shock) and their unobservable health characteristics.

Control for individual heterogeneity is achieved through two channels. First, the calendar year when individuals are exposed to an initial health shock has some degree of randomness with respect to the technology and medical practice available to treat them after the shock. For instance, hospitals decision to invest in acute care for patients with an AMI or a stroke in a particular year has little influence on the probability that individuals will suffer from an AMI or a stroke in that year. Second, we use a large basket of indicators controlling for individual heterogeneity in health and socioeconomic characteristics over the calendar years when individuals are exposed to the health shock. For instance, a health shock might be more severe in late calendars years if the population becomes older and sicker, or if the health system rises the bar for an emergency hospital admission. However, the extent of this potential bias should not be large given the relatively short time window examined by our study, and it should be possible to control for by using the large basket of individual indicators at our disposal. We provide two tests for the extent of such a potential bias in the section for robustness checks.

We attribute the RI of HCE captured by Equation (1) to the overall change in technology and medical practice that occurred during the examined time, *t*, following a similar interpretation in macroeconomic studies using a residual approach (Chernew & Newhouse, 2011). Our identification assumption is that the calendar year when an individual experience an initial health shock and the severity of the shock are independent from technological progress in hospital acute care after controlling for observable individual heterogeneity. We provide evidence supporting this assumption in the robustness‐checks section of the paper.

### 3.2 Decomposing the RI of HCE

Equation (1) provides a snapshot of the RI of HCE with respect to the *calendar time* when individuals are exposed to a health shock. However, the RI of HCE can be also examined with respect to the *elapsed time* from the health shock as the difference in two cost accumulation functions produced by individuals who are exposed to a health shock in two different calendar years. In other words, we can study the trajectory followed by the RI from the time point when the health shock onsets to different end points of interest, for example, 30 days, 180 days, 365 days or 1095 days after the shock, and examine how the RI of HCE accumulates over such time windows. To this end, we apply the estimator proposed by Basu and Manning (2010) that extends the class of two‐part models to deal with spikes in cost‐accumulation due to TTD. The BM‐estimator allows us to decompose the RI in the part that is due to increasing survival, that is, the *delaying TTD effect*, and the part that is due to increasing resource use per unit of time, that is, the *intensity effect*. Both effects are likely to occur as a result of investments in new technologies and medical practices improving quality of care and reducing hospital mortality rates for many health conditions (OECD et al., 2017). The delaying TTD effect is the result of patients surviving longer and thus contributing longer to the demand of care. The intensity effect is the net results of distinct sources of variation in HCE: on one hand the basket of services accessed by patients becomes more expensive over time to allow for the cost of innovation; on the other hand, new technology and medical practice may reduce the use of unnecessary care, for example, emergency hospital readmissions, and allow policy makers to redirect the demand to less expensive and equally effective level of care,^6^ for example, from inpatient to outpatient care, incentivizing productivity (OECD et al., 2017).

Basu and Manning (2010) propose the following model to describe the process of cost accumulation for an individual *i* over a number of discrete time periods *j* = *1 …*, *K*:

(2) $$\mu =\sum_{j=1}^{K}\mathrm{Pr}\left(V>a_{j-1}\right)*\left\{\mu_{1j}*h\left(a_{j}\right)+\mu_{2j}*\left(1-h\left(a_{j}\right)\right)\right\}$$

Where μ is the expected cumulative HCE up to the period *j* = *1*,…, *K* for the individual *i* (the notation for individuals has been suppressed for clarity); $h\left(a_{j}\right)$ is the hazard of death in the interval $\left(a_{j},\;a_{j-1}\right]$ for individuals who survived until $a_{j-1}$; $\mathrm{Pr}\left(V>a_{j}\right)=S\left(a_{j}\right)$ is a survival function for the individual *i* with *V* indicating her TTD. An appealing property of this model is that the rate of cost accumulation in individuals who die is allowed to differ from individuals who do not, with $\mu_{1j}$ representing the expected HCE if the subject dies in the interval *j* and $\mu_{2j}$ the expected HCE if she survives.

The model in Equation (2) can be estimated using a three‐part estimator over different subsets of person‐period observations: *Part‐1* estimates the predicted probability of survival $\hat{S_{j}}\left(X\right)$ until the start of the period *j* and the hazard function for death during the period $\hat{h_{j}}\left(X\right)$ for all person‐period observations. We estimated Part‐1 with a pooled logit model using a discrete‐time approach that allows us to estimate: (a) the time‐varying effects, which enter the model through the interaction between the periods *j* = 1,…, K and capture the time elapsed after the health shock; and (b) the calendar time *t* = 1,…,T capturing the RI of HCE. *Part‐2* estimates ${\hat{\mu }}_{1j}\left(X\right)$ in the person‐periods in which individuals die, and *Part‐3* estimates ${\hat{\mu }}_{2j}\left(X\right)$ in the person‐periods in which the individual survives. We estimated Part‐2 and Part‐3 by using a two‐part model with the first part consisting in a Logit model for the probability of positive HCE and the second part consisting in a GLM model for positive HCE with gamma distribution and log link function. The estimated cost function for an interval j for any individual can be expressed as:

(3) $$\hat{\mu_{j}}\left(X\right)=\hat{S_{j}}\left(X\right)*\left[\hat{h_{j}}\left(X\right)*{\hat{\mu }}_{1j}\left(X\right)+\left(1-\hat{h_{j}}\left(X\right)\right)*\;{\hat{\mu }}_{2j}\left(X\right)\right]$$

with: $\hat{µ}\left(X\right)=\sum_{j=1}^{K}\hat{\mu_{j}}\left(X\right)$

Formal proof of the consistency of the estimator is in Basu and Manning (2010). Equation (3) allows us to model two dimensions of time that describe the data generating process. First, the calendar time *t* when individuals experience the health shock, which we include in the matrix of covariates X as a vector of dummies.^7^ Here, the calendar time *t* captures the RI of HCE that can be attributed to technological progress and medical practice following a similar approach to the model in Equation (1). Second, the time elapsed after the health shock *j* (with *j* = *1*, *2*, *… K*) that we capture by estimating the HCE for each person‐period observation after the shock, $\hat{\mu_{j}}\left(X\right)$, and then cumulating the sum of $\hat{\mu_{j}}\left(X\right)$ from the first to the last of the periods *j*.

The RI of HCE, which can be attributed to the overall change in technology and medical practice, is obtained by differentiating Equation (3) with respect to calendar years Δt ^8^:

(4) $$\begin{array}{ccc} \frac{\Delta \mu \left(X\right)}{\Delta t} & = & \sum_{j=1}^{K}\left\{\frac{\Delta \hat{S_{j}}\left(X\right)}{\Delta t}\left[\hat{h_{j}}\left(X\right)*{\hat{\mu }}_{1j}\left(X\right)+\left(1-\hat{h_{j}}\left(X\right)\right)*{\hat{\mu }}_{2j}\left(X\right)\right]+\hat{S_{j}}\left(X\right)\left[\frac{\Delta \hat{h_{j}}\left(X\right)}{\Delta t}*\left({\hat{\mu }}_{1j}\left(X\right)-{\hat{\mu }}_{2j}\left(X\right)\right)\right]\right\}+\\ & & +\left\{\hat{S_{j}}\left(X\right)\left[\hat{h_{j}}\left(X\right)*\frac{\Delta {\hat{\mu }}_{1j}\left(X\right)}{\Delta t}+\left(1-\hat{h_{j}}\left(X\right)\right)*\frac{\Delta {\hat{\mu }}_{2j}\left(X\right)}{\Delta t}\right]\right\} \end{array}$$

The first part of Equation (4) in curly brackets captures the RI of HCE that is due to a change in the probability of surviving between the two calendar years, that is, the delaying TTD effect, while the second part captures the RI of HCE that is due to a change in the rate of cost accumulation per unit of time, that is, the intensity effect. The delaying TTD effect is measured for an expected level of HCE predicted by ${\hat{\mu }}_{1j}\left(X\right)$ and ${\hat{\mu }}_{2j}\left(X\right)$ for every subject‐intervals in the data (first part of Equation 4); similarly, the intensity effect is measured for an expected level of survival predicted by $\hat{S_{j}}\left(X\right)$ (second part of Equation 4). Finally, the exponential trajectory of HCE in individuals entering TTD is modeled by estimating two distinct cost functions, ${\hat{\mu }}_{1j}\left(X\right)$ and ${\hat{\mu }}_{2j}\left(X\right)$, for the *j* person‐periods in which individuals die and in which they survive.

Notice that under this framework, individual characteristics and health care resources included in *X* are assumed to have a distinct and independent effect on individual survival, $\hat{S_{j}}\left(X\right)$, and cost of care ${\hat{\mu }}_{1j}\left(X\right)$ and ${\hat{\mu }}_{2j}\left(X\right)$. This is a key property of the cost function in Equation (2) that allows us to capture the effect of technological progress and change in medical practice, Δt, on HCE through a distinct and independent effect on TTD and intensity of resource use. This approach has been used for measuring the effect of alternative treatments on cost trajectories (Federspiel et al., 2013; White et al., 2019; Williams et al., 2019) and in the formulation of other cost functions (Lin, 2003). However, other studies adopt a different modeling solution in which the survival function, or the TTD indicator, enters the cost function as an independent variable together with individual characteristics and health care resources (de Meijer et al., 2011; Howdon & Rice, 2018).

## 4 RESULTS

Table 2 reports estimates of RI of HCE from the GLM model described in Equation (1) in the method section. HCE is measured as the total amount paid for inpatient and outpatient services accessed by individuals in our study from the onset of the health shock to 365 days after. The RI is captured by the calendar year dummies under five different model specifications with increasing number of covariates testing its sensitivity to variation in age, morbidity, and socioeconomic status. We use 2‐year intervals to allow for a minimum of 10 observations in each morbidity group. The average HCE per person increased by 16.90% points in individuals experiencing an initial health shock in 2013‐14 as compared to similar individuals in 2005‐6 (Model 1). After controlling for variation in age and morbidity and socioeconomic status, the increment in HCE between the 2 periods drops to 10.28% points (Model 5), namely the estimated RI of HCE that can be attributed to technological progress and change in medical practice accounts for about 60% of the total increment of HCE (i.e., 10.28/16.90 = 60.82%). This result is in the ballpark of macroeconomic studies measuring the impact of technological progress on HCE using a residual approach (Newhouse, 1992; Peden & Freeland, 1998; Smith et al., 2009). Variation in age accounts only for about 7% of total increment (i.e., 1–15.77/16.90 = 6.87; Model 2). Morbidity and age together account for about 40% of total increment (i.e., 1–10.19/16.90 = 39.70; Model 4), while including variation in socioeconomic status leave the total increment unchanged (Model 5). The latter result is not surprising as the DNHS is a universal health system with access to care independent from income. This analysis suggests that variation in age plays a limited role in explaining variation in HCE over time, while variation in morbidity has a much larger influence as found by other studies (de Meijer et al., 2011; Howdon & Rice, 2018; Moore et al., 2017; Shang & Goldman, 2008). However, both age and morbidity are blunt predictors of the increment of HCE over time as about 60% of its total increment remains unexplained after allowing for these factors.

**TABLE 2:** Residual Increment of annual HCE in individuals with an initial health shock

	Model 1	Model 2	Model 3	Model 4	Model 5
Shock 2005‐6	Baseline	Baseline	Baseline	Baseline	Baseline
Shock 2007‐8	0.9894**	0.9881***	0.9830***	0.9792***	0.9794***
	(0.0043)	(0.0042)	(0.0038)	(0.0038)	(0.0038)
Shock 2009‐10	1.0700***	1.0648***	1.0389***	1.0307***	1.0312***
	(0.0047)	(0.0047)	(0.0042)	(0.0041)	(0.0041)
Shock 2011‐12	1.1210***	1.1125***	1.0793***	1.0684***	1.0690***
	(0.0050)	(0.0049)	(0.0044)	(0.0043)	(0.0043)
Shock 2013‐14	1.1690***	1.1577***	1.1191***	1.1019***	1.1028***
	(0.0052)	(0.0051)	(0.0046)	(0.0044)	(0.0045)
Female		0.8733***	0.9147***	0.9126***	0.9108***
		(0.0025)	(0.0025)	(0.0025)	(0.0025)
Age		1.1274***	1.0869***	1.0849***	1.0854***
		(0.0017)	(0.0015)	(0.0014)	(0.0015)
Age sq.		0.9991***	0.9994***	0.9994***	0.9994***
		(0.0000)	(0.0000)	(0.0000)	(0.0000)
Total diagnoses			1.1294***	1.1190***	1.1187***
			(0.0012)	(0.0012)	(0.0012)
Charlson index			1.2220***	1.1769***	1.1767***
			(0.0145)	(0.0136)	(0.0136)
15 comorbidities indicators			Yes	Yes	Yes
175 primary diagnosis indicators			Yes	No	No
1005 primary diagnosis indicators			No	Yes	Yes
Single					1.0139***
					(0.0028)
Migrant					0.9401***
					(0.0056)
Income					1.0000
					(0.0000)
Income sq.					1.0000
					(0.0000)
HCE 1 year before the shock			1.0012***	1.0012***	1.0012***
			(0.0000)	(0.0000)	(0.0000)
HCE 2 years before the shock			1.0006***	1.0006***	1.0006***
			(0.0000)	(0.0000)	(0.0000)
Constant	15,004.8381***	272.1890***	402.0070***	1249.3888***	1295.9371***
	(45.6520)	(14.2397)	(20.5177)	(732.8990)	(774.3949)
Observations	962,794	962,794	962,794	962,794	962,794
BIC‐	12203624	−12221661	−12432801	−12457751	−12457898

*Note*: Exponentiated coefficients from GLM regression. Robust SE in parentheses.

Abbreviations: BIC, Bayesian information criterion; GLM, generalised linear model; HCE, health care expenditure.

***p < 0.01, **p < 0.05, *p < 0.1.

Table 3 reports results of the decomposition analysis of the RI into the part that is due to delaying TTD and the part that is due to increasing intensity of resource use. Estimates are obtained from the BM‐estimator described in Equations (2)–(4).^9^ HCE is measured as the *cumulated* total amount paid for inpatient and outpatient services accessed by individuals in our study from the onset of the health shock to 365 days, 730 days, and 1095 days after. The RI is captured by the difference in HCE for individuals having a health shock in 2013‐14 as compared with 2005‐6 after controlling for variation in age, morbidity and socioeconomic characteristics using the same parametrization of Model 4 in Table 2. The RI is measured as a differential effect (i.e., average marginal effect) and cumulated over the time elapsed from the health shock up to 3 years after. Standard errors are calculated from 500 clustered bootstrap replicates. Figure 3 plots results in Table 3 against the time elapsed from the health shock providing more granularity to the trajectories of cost accumulation. Three years from the health shock, the cumulated total RI f HCE that can be attributed to technological progress and change in medical practice amounts to 2006 € per patient (at 2017 price level); about one fourth of the total RI (508 €) is due to delaying TTD, that is, individuals using more health care resources as they survive a health shock for longer than their peers in 2005‐6, while the rest of the RI is due to increasing intensity of resource use (1498 €), that is, individuals consuming more health care resources per unit of time. Finally, Table 3 and Figure 3 show that about 63% of the total RI is produced in the first 365 days after the health shock (1278 €), while the remaining 37% is accumulated in year 2 and year 3 from the health shock in equal shares.

**TABLE 3:** Residual increment of HCE from 2005‐6 to 2013‐14

	0–365 days after shock			0–730 days after shock			0–1095 days after shock		
	RI total	RI due to delaying TTD	Ri due to intensity of resource use	RI total	RI due to delaying TTD	RI due to intensity of resource use	RI total	RI due to delaying TTD	RI due to intensity of resource use
All									
2005‐26	Baseline	Baseline	Baseline	Baseline	Baseline	Baseline	Baseline	Baseline	Baseline
2007‐8	−0.121	0.022	−0.143	0.118	0.058	0.060	0.324	0.082	0.243
S.E	(0.057)	(0.010)	(0.058)	(0.077)	(0.019)	(0.078)	(0.089)	(0.023)	(0.090)
2009‐10	0.583	0.085	0.497	1.154	0.209	0.945	1.526	0.283	1.243
S.E.	(0.057)	(0.010)	(0.057)	(0.084)	(0.019)	(0.082)	(0.100)	(0.024)	(0.097)
2011‐12	1.031	0.120	0.911	1.789	0.278	1.511	2.233	0.366	1.868
S.E.	(0.057)	(0.009)	(0.058)	(0.078)	(0.018)	(0.080)	(0.091)	(0.021)	(0.093)
2013‐14	1.278	0.174	1.104	1.800	0.392	1.408	2.006	0.508	1.498
S.E.	(0.063)	(0.009)	(0.063)	(0.085)	(0.019)	(0.084)	(0.097)	(0.023)	(0.097)
Cancer									
2005‐6	Baseline	Baseline	Baseline	Baseline	Baseline	Baseline	Baseline	Baseline	Baseline
2007‐8	−1.140	0.988	−2.128	−0.975	1.565	−2.540	−0.887	1.766	−2.653
S.E.	(0.408)	(0.217)	(0.384)	(0.554)	(0.325)	(0.511)	(0.624)	(0.359)	(0.576)
2009‐10	1.125	1.641	−0.516	1.925	2.676	−0.750	2.239	3.058	−0.819
S.E.	(0.433)	(0.232)	(0.409)	(0.586)	(0.343)	(0.537)	(0.657)	(0.377)	(0.603)
2011‐12	3.089	2.136	0.953	4.602	3.418	1.184	5.162	3.873	1.289
S.E.	(0.412)	(0.227)	(0.394)	(0.560)	(0.345)	(0.510)	(0.635)	(0.382)	(0.574)
2013‐14	4.134	2.667	1.467	5.155	4.253	0.901	5.326	4.810	0.515
S.E.	(0.442)	(0.239)	(0.438)	(0.564)	(0.364)	(0.554)	(0.614)	(0.404)	(0.606)
AMI									
2005‐206	Baseline	Baseline	Baseline	Baseline	Baseline	Baseline	Baseline	Baseline	Baseline
2007‐8	−0.316	0.041	−0.357	−0.068	0.104	−0.172	0.096	0.124	−0.028
S.E.	(0.270)	(0.028)	(0.271)	(0.358)	(0.066)	(0.358)	(0.406)	(0.078)	(0.406)
2009‐10	−0.263	0.070	−0.333	0.430	0.192	0.238	0.872	0.237	0.635
S.E.	(0.293)	(0.027)	(0.295)	(0.405)	(0.061)	(0.406)	(0.469)	(0.072)	(0.469)
2011‐12	−0.238	0.082	−0.320	0.394	0.228	0.167	0.787	0.280	0.506
S.E.	(0.302)	(0.028)	(0.305)	(0.401)	(0.064)	(0.407)	(0.457)	(0.076)	(0.463)
2013‐14	−1.465	0.141	−1.606	−1.093	0.382	−1.476	−0.746	0.467	−1.213
S.E.	(0.315)	(0.029)	(0.317)	(0.423)	(0.063)	(0.423)	(0.477)	(0.074)	(0.476)
Stroke									
2005‐6	Baseline	Baseline	Baseline	Baseline	Baseline	Baseline	Baseline	Baseline	Baseline
2007‐28	−0.003	0.005	−0.008	0.369	0.019	0.350	0.603	0.026	0.577
S.E.	(0.266)	(0.043)	(0.266)	(0.369)	(0.082)	(0.366)	(0.433)	(0.094)	(0.429)
2009‐10	2.257	0.037	2.219	3.070	0.109	2.961	3.364	0.144	3.220
S.E.	(0.331)	(0.045)	(0.329)	(0.421)	(0.087)	(0.415)	(0.465)	(0.101)	(0.458)
2011‐12	3.330	0.115	3.215	4.619	0.269	4.350	5.097	0.332	4.765
S.E.	(0.349)	(0.049)	(0.352)	(0.451)	(0.097)	(0.455)	(0.504)	(0.113)	(0.509)
2013‐14	6.249	0.181	6.068	7.620	0.392	7.228	7.878	0.471	7.407
S.E.	(0.412)	(0.043)	(0.412)	(0.517)	(0.082)	(0.513)	(0.560)	(0.094)	(0.555)

*Note*: Total residual increment and decomposition into delaying time to death effect and intensity effect. (1000 €). Estimated average marginal effects from Basu‐Manning estimator in Equation (4). All models include controls for variation in age, morbidity and socioeconomic characteristics using the same specification of Model 4 in Table 2. Standard errors calculated from 500 clustered bootstrap replicates.

Abbreviations: AMI, acute myocardial infarction; HCE, health care expenditure; RI, residual increment; TTD, time to death.

**FIGURE 2:**
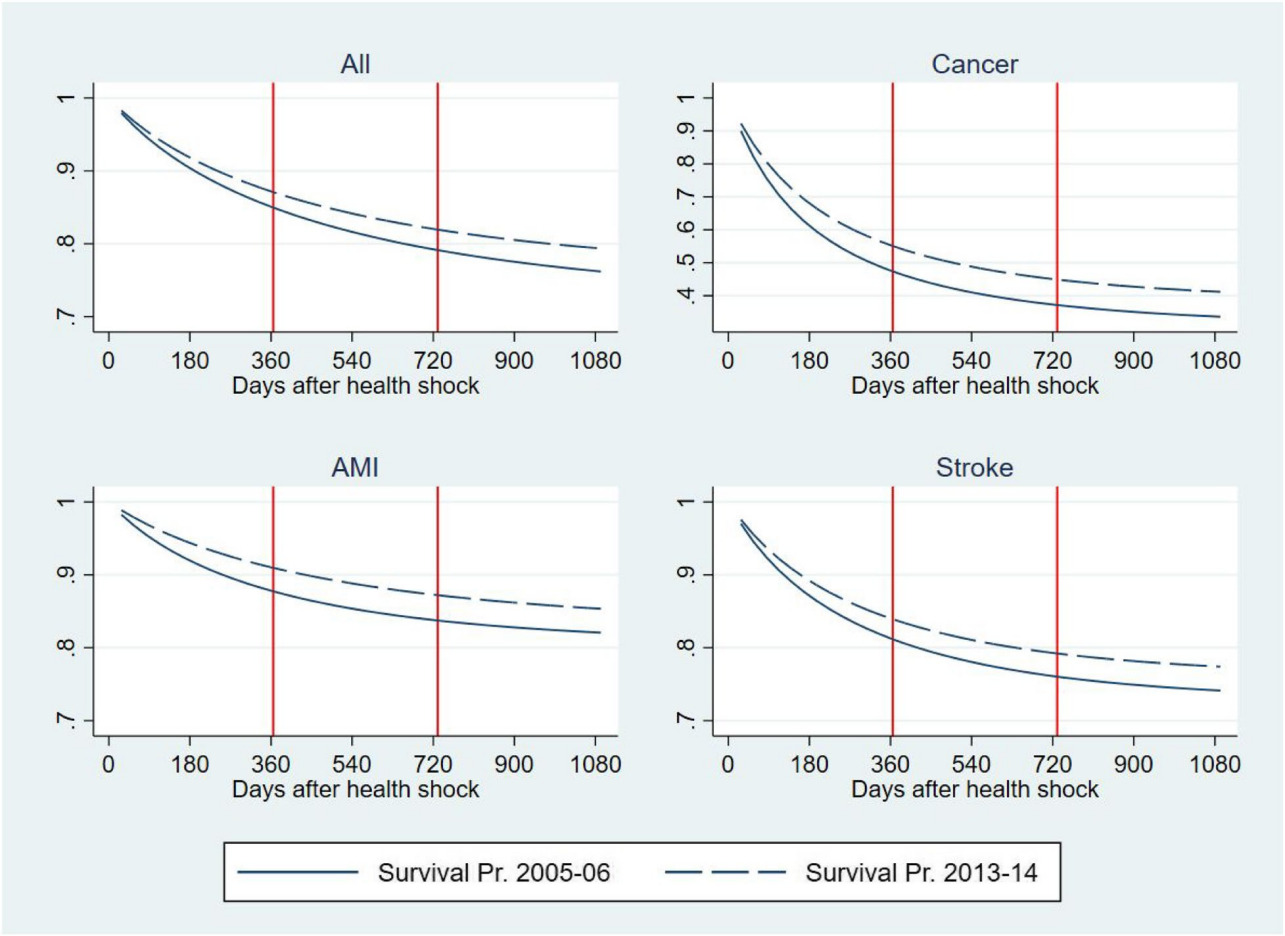
Cumulative survival probability after a health shock in 2013‐14 and 2005‐6. Predictions from a logit model

Figure 2 reports the predicted cumulative survival distribution for individuals having an initial health shock in 2013‐14 and 2005‐6 over the following 3 years. Predictions are obtained from the logit model used to estimate Part 1 of the three‐part BM‐estimator described in the method section.

**FIGURE 3:**
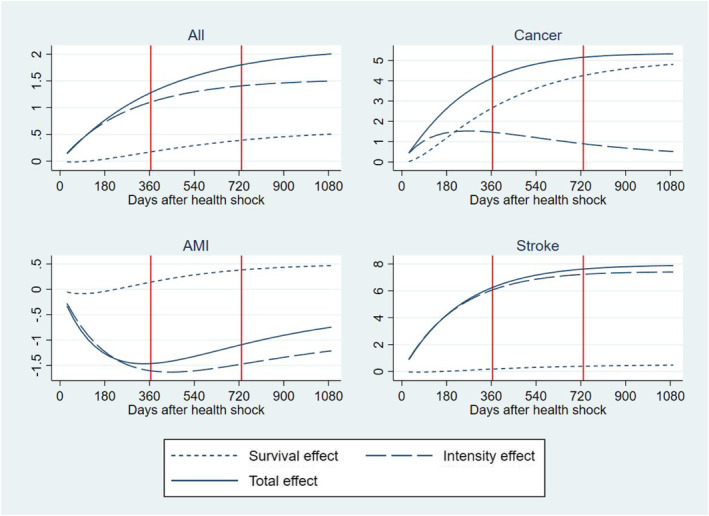
Residual increment of health care expenditure in 2013‐14 versus 2005‐6 (baseline). Total increment (continuous line) and decomposition into delaying time to death effect (dotted line) and intensity effect (dashed line). Cumulative distribution over the time elapsed from initial health shock. (*Y* = 1000 €)

Table 3 and Figure 3 also report results of the heterogeneity analysis by health conditions prompting the initial health shock, that is, the health condition reported as the primary diagnosis at the time of the health shock. We examined three acute conditions that gained noticeable increments in their survival rates during the time of our study (OECD et al., 2017): cancer, AMI, and strokes. With respect to cancer, we examined the four most prevalent type of cancer (colorectal, breast, prostate and lung) and included controls for cancer site and metastatic cancer in the regression. As expected, the RI and its components show a large heterogeneity across these conditions as they attract different treatments with associated different trajectories of technological progress and medical practice. In cancer, the cumulated total RI per patient is two and a half times larger than in the total population amounting to a total of 5326 € 3 years after the shock; about 77% of this total is produced in the first year after the shock and about 90% is due to delaying TTD. Individuals in this group include newly diagnosed patients during an emergency admission and formerly diagnosed patients with a deterioration in health conditions leading to an emergency admission, both attracting a high mortality risk and amount of resources (Laudicella, Walsh, et al., 2018). The large share of the RI due to the delaying TTD effect can be explained by a large improvement in survival outcomes (Figure 2) and a relatively small increment in the average amount of resources allocated to patients after an initial shock due to cancer, that is, a large $\Delta \hat{S_{j}}$ and a small $\Delta \hat{\mu_{j}}$ in Equation (4). Moreover, cancer patients consume a high level of resources, that is, a large ${\hat{\mu }}_{j}$ (Equation 4), which amplifies the share of the RI that the cost function apportions to the survival effect.

The cumulated total RI for strokes amount to 7878 € per patient 3 years after the shock; similarly to cancer, a large share of this total is produced in the first year after the shock (74%), but more than 90% is due to increasing intensity of resource use, rather than delaying TTD. This can be explained by a smaller improvement in survival outcomes as compared to cancer (Figure 2) and a relatively larger increment in resources allocated to these patients, including timely intervention for patients living in rural areas and rehabilitation services provided in hospital outpatient setting.

Finally, the RI for AMI is negative suggesting a saving of −1465 € after the first year from the health shock and reducing to −746 € after 3 years. The latter is the result of a large reduction in intensity of resource use and a small increment in survival effect over time. This might be explained by an increment in the offer of rehabilitation services for cardiac patients in Denmark, which reduced the risk of re‐hospitalization and total number of bed days during the period of our study, and by a shift of part of the rehabilitation services from the hospital to the municipality (Lindstrom Egholm et al., 2018).

### 4.1 Robustness checks

We tested the robustness of our findings to potential bias from unobservable individual heterogeneity and measurement errors. First, we compared estimates of the RI of HCE obtained from 2 different baskets of primary diagnosis indicators: the first basket consisted of 1005 indicators based on the first 3 digits of the ICD‐10 code (Model 5, Table 2), while the second basket consisted of 175 indicators obtained from the first 2 digits of the former (Model 4, Table 2). In other words, we compared estimates obtained by using the basket of most accurate control indicators at our disposal with estimates obtained by reducing the level of accuracy of such indicators. If results are robust to such a large drop in the level of accuracy of our control indicators, then we can expect a small scope for bias from unobservable individual heterogeneity and coding error in individual diagnosis. We found that estimates of the RI are about one percentage point smaller when using the more accurate basket of indicators suggesting that the scope for potential bias is small.

Second, we extended model in Equation (1) by including a longitudinal dimension:

(5) $$E\left(HCE_{ij}|x_{ij}\right)=\exp\left(t_{j}+\beta X_{ij}\right)$$

with *j* defining the longitudinal dimension of time as the time elapsed after a health shock for the individual *i*, while *t* capturing the calendar time when the shock occurred. We estimated the RI of HCE from Equation (5) by using a population average Generalized Estimating Equations (GEE) with gamma distribution and log link function, which allows for within‐individual dependence of observations. We considered *j* = 1, 2, 3 years after the health shock. Equation (5) provides an alternative strategy to control for individual heterogeneity that might confound the identification of the RI of HCE by using individual random effects. The GEE model is consistent under the correct specification of the mean function and does not require distributional assumptions on the individual random effects of the mixed models. The GEE model was estimated using the same specification of Model 4 in Table 2, as it did not reach convergence under the full model specification of Model 5. Point estimates differ by less than one percentage point with respect to estimates produced by the GLM under Model 5 specification^10^ (Equation 1 and Table 2).

Third, we repeated our analysis in a subsample of the study population with a health shock resulting in at least 2 days of hospital length of stay (Appendix 2). This means selecting individuals with more severe health shocks and dropping about one third of the original study population as many emergency admissions have a length of stay of just 1 day. In this subsample, the increment in HCE that can be attributed to variation in technological progress and change in medical practice is 54.72% as compared with 60.82% in the total population, which leads to similar conclusion to our main analysis. However, the total increment of HCE over the period of our study is larger in the subsample (23.96%) as compared with the total population (16.90%).

Finally, survival probabilities predicted from the logit model to estimate Part‐1 of the BM‐estimator are similar to predictions obtained by using a non‐parametric Kaplan‐Meier estimator.

## 5 DISCUSSION

This study uses a residual approach and microdata to measure the impact of non‐demographic and non‐health related drivers of the HCE, such as technological progress and change in medical practice. We focus on residents of Denmark experiencing an initial health shock from 2005 to 2014 and accounting for 23.5% of the national HCE for hospital inpatient and outpatient care. During this period, HCE per patient increased by 16.90% points in real terms. We found that 60% of such an increment is not explained by variation in morbidity or socioeconomic factors and can be attributed to technological progress and change in medical practice in hospital care. Macroeconomic studies using a residual approach (Newhouse, 1992; Peden & Freeland, 1998; Smith et al., 2009) and also a direct approach with indicators of investment in R&D as a proxy for technological progress (Okunade & Murthy, 2002; Willemé & Dumont, 2015) reach similar conclusions, estimating an impact between 40 and 70% on HCE growth. The residual approach has the advantage of bypassing the adoption of a specific definition of technological progress and indicators capturing it; both pose a long standing challenge to research on this topic as technological progress has different meanings for different sectors of health care and encompasses heterogeneous aspects that are often difficult to capture by existing indicators, for example, investment in R&D, patents for new drugs and medical devices, hospital investments (Chernew & Newhouse, 2011). The RI of HCE captures a broad definition of technological progress described in Chernew and Newhouse (2011), including innovation that results in new products and services, innovation that results in new applications of existing products and services, and process innovation that results in lowering production costs by changing the organization of the production and delivery of existing products and services. The latter is likely to be responsible for the drop in the RI of HCE in 2007‐8 after the introduction of a reform that reorganized the hospital sector in Denmark creating large multi‐service organizations and centralizing specialized services (Christiansen & Vrangbaek, 2018). Disentangling the different channels of technological progress would require new methods and data and should be a subject of future research.

Applying a residual approach to microdata allows us to provide an accurate control for variation in HCE that is due to morbidity and to avoid assumptions over factors encouraging technological progress, such as income elasticity. Both are often problematic variables in macroeconomic models as the former is often unavailable at the macro level and the latter influences the predicted effect of technological progress on HCE (Chernew & Newhouse, 2011). However, our identification of the impact of technological progress relies on controlling for observable characteristics of the demand that influence HCE over time. Although we use a large basket of control indicators for individuals' health and socioeconomic characteristics, we are unable to control for large exogenous shock to the demand. For instance, a strong economic shock, such as the 2008 financial crisis, may generate a temporary increment in the demand of some health services, for example, mental health care, in individuals with similar health and socioeconomic conditions to pre‐crisis periods; similarly, a new health shock in a large section of the population, such as a pandemic disease, may disrupt the demand and supply of some health services in the medium term. In these cases, the identification of the impact of technological progress using a residual approach may be difficult to achieve. Moreover, our specific application does not include utilization of primary care and pharmaceuticals prescribed by the GP. If some hospital services are shifted to primary care over time, this may result in underestimating the RI and thus the impact of technological progress (in hospital care) on HCE. However, this is not an inherit limitation of our approach and primary care costs can be included in future works.

Using microdata and the BM‐estimator allow us to decompose the impact of technological progress on HCE in the part that is due to delaying TTD and the part that is due to the intensity of resource use. Technological progress and change in medical practice are likely to produce their impact on HCE through two main channels: first individuals are able to survive a health shock for a longer time, hence they can continue to contribute to the demand for health care during that time; second the basket of health care services they access is likely to become more expensive to allow for the cost of innovation. The decomposition exercise provides useful information on the impact of technological progress on HCE over time. It allows the researcher to apportion the contribution of delaying TTD and increasing intensity of resource use consistently and examine their impact over time separately. A pure intensity effect may occur if technological progress has no impact on survival; similarly, a pure survival effect may occur if technological progress has no impact on intensity of resource use. Both cases are possible. For instance, the intensity effect is likely to include savings in HCE from reducing the use of unnecessary care, for example, emergency hospital readmissions, and from redirecting the demand to less expensive care, for example, from inpatient to outpatient care; both could result in a zero or negative intensity effect. Disentangling the different components that contribute to the intensity effect goes behind the scope of this study and should be the object of further work.

The identification of a distinct effect of technological progress on HCE through the survival effect and the intensity effect is achieved from the functional form of the cost function modeling HCE (Equation 2), rather than from a distinct source of variation of technological progress affecting survival and intensity of resource use separately. The latter would be quite difficult to achieve as survival and use of resources are intertwined processes for many health conditions, that is, patients are able to survive as they use health resources and are able to use health resources as they survive. The cost function of the BM‐estimator assumes that health care resources have a distinct and independent effect on survival and costs that ultimately allows for decomposing the marginal effect of technological progress into a survival and an intensity effect. A growing number of studies have adopted a similar cost function and decomposition approach for measuring the effect of alternative treatments on cost trajectories (Federspiel et al., 2013; White et al., 2019; Williams et al., 2019). In contrast, a common approach in modeling the effect of TTD on HCE is to include the former directly in the cost function as an independent variable (de Meijer et al., 2011; Howdon & Rice, 2018).

We found evidence that delaying TTD can explain one quarter of the increment in HCE 3 years after the onset of the initial health shock, while the remaining part can be attributed to an increment in intensity of resource use. The former can be considered a “side effect” of the success of the health system in improving quality of care and reducing mortality rates as shown in this study and elsewhere (Laudicella, Martin, et al., 2018). Although we cannot exclude that the variation in the probability of surviving over time is also affected by other processes, such as increasing longevity due to the slowing down of the process of aging, the latter is likely to play a minor role due to the relatively short interval of time and the specific population examined in this study. Finally, the magnitude of the delaying TTD effect is likely to change over time. If future technological advances entail lower costs of treatment, than the cost of individuals surviving longer will decrease.

Our study contributes to the literature on TTD and HCE. Macroeconomic models show that postponing TTD results in reducing HCE growth as the high costs associated with TTD are moved forward to future periods (Polder et al., 2006; Stearns & Norton, 2004; Wickstrøm et al., 2002). More sophisticated models show that the cost‐saving effect of postponing TTD is off‐set by an increment in costs due to “unspecified causes”, normally attributed to technological progress in the literature (van Baal & Wong, 2012). In our study framework, the RI captures “unspecified causes” of HCE growth using micro data, and the effect of delaying TTD in the first part of our model (Equation 4) can be offset by the intensity effect in the second part. Postponing TTD may result in reducing individual HCE if the person‐periods in which individuals die are moved forward outside the time window examined by the study, that is, the first 3 years after the health shock. However, our empirical application focuses on a population exposed to a health shock with a large share of mortality events occurring within a close range from the shock. In our study population, the effect of postponing TTD is to increase HCE as a large share of high‐risk individuals enter TTD within the examined time window, namely their TTD is just delayed. Such a prediction differs from studies on TTD showing that postponing TTD may have a cost reducing effect on HCE. This is not surprising as we examine mortality in a subgroup of the population exposed to a health shock, rather than in the whole population. The choice of this specific setting allows us to model variation in TTD as an outcome of technological progress in hospital care, while most of the existing literature includes variation in TTD as an exogenous variable. The relationship between postponing TTD and HCE is likely to be different in other subgroups of the population for whom gains in life expectancy are the outcome of slowing down the process of aging, for example, by assuming healthy life styles and behaviors, or the outcome of technological progress outside the hospital sector, for example, in preventive care.

The period examined in our study encompasses a reform of the hospital sector that created large multi‐service organizations and centralized specialized services. Undoubtedly, this reform contributed to the trajectory of HCE in Denmark. However, we argue that such a policy intervention should not be considered as a confounding effect to the identification of the effect of technological progress and change in medical practice, rather it is one of the forces that lead these two processes in the health system. Other forces include physicians' beliefs and patient preferences (Cutler et al., 2019), the structure of the pharmaceutical market (Acemoglu & Linn, 2004), and the diffusion and coverage of health insurance (Finkelstein, 2007). Publicly funded universal health systems, such as the DNHS, are subject to heavy regulation offering a limited scope to market forces for leading changes in the system, hence technological progress and change in medical practice are often made possible through policy interventions. Disentangling the contributions of different forces in conveying technological progress and their impact on HCE should be the object of further work.

## CONFLICT OF INTEREST

The authors disclose no conflict of interest.

## ETHICS STATEMENT

The analysis involved the use of previously collected and pseudonomised individual level data on access to health services. Ethics approval to conduct the study has been granted by Statistics Denmark (DST) and the University Research and Ethics Commission.

## Supporting information

Supplementary Material 1

Supplementary Material 2

## Data Availability

The data that support the findings of this study are available upon submission of an application to Statistics Denmark, https://www.dst.dk/en. Restrictions may apply to the availability of these data, which were used under license for this study.

## ENDNOTES

^1^
Health shocks are more likely to occur in individuals age 50+ than in the rest of the population.

^2^
Using health shocks as an exogenous source of variation has been used in the literature, see Garcia‐Gomez et al., 2013, Fadlon and Nielsen, 2021.

^3^
The analysis is robust to using higher thresholds, such as 2 days.

^4^
The accuracy of the inflation index is important for removing variation in the price of hospital services unrelated to variation in real inputs and outputs over time, and thus for the correct estimation of the RI of HCE.

^5^
We also conducted a longitudinal analysis based on the time elapsed after the health shock, which is descried in the robustness checks section of the paper

^6^
In decomposing the increment of HCE, de Meijer et al. (2013) give a similar interpretation to the variation in the effect of the drivers of HCE over time as the net effect of both new technologies and new policies.

^7^
The covariates included the matrix *X* are measured in the calendar year *“t”* when individuals experience an initial health shock and can vary over the time *“j”* elapsed after the shock; the vector of calendar years *“t”* is fixed.

^8^
In our empirical application, we calculate population average marginal effects for *t* = 2005‐6 (baseline year) against *t* = 2007‐8, 2009‐10, 2011‐12, and 2013‐14.

^9^
Appendix 1 reports the full list of output tables and estimated parameters of the Basu‐Manning estimator. Estimates were obtained using Stata version 16.

^10^
Estimates of the GEE model are available upon request from the authors
