# Supplementary material for: Age, morbidity, or something else? A residual approach using microdata to measure the impact of technological progress on health care expenditure
Source: Health Econ. 2022 Mar 31;31(6):1184–201. doi: 10.1002/hec.4500 (PMC9314678; doi:10.1002/hec.4500)
Supplement: Supplementary file 1 — Supplementary Material 1 [file HEC-31-1184-s002.docx]

**Appendix 1**

Below, we report output tables and estimated parameters of the Basu-Manning estimator described in Eq. 3 for our study population. In order to estimate the model, data have been transformed in person-period format.

**Part-1** estimates the predicted the probability of survival $\hat{S}_{j}(X)$. This is achieved by estimating a pooled logit model using a discrete-time approach on the probability of dying. Results are reported in the regression output below.

Logistic regression Number of obs = 31,816,056

LR chi2(216) = 405950.97

Prob > chi2 = 0.0000

Log likelihood = -1109942.9 Pseudo R2 = 0.1546

---------------------------------------------------------------------------------------

deadperiod | Coef. Std. Err. z P>|z| [95% Conf. Interval]

----------------------+----------------------------------------------------------------

diagtot_0 | .0868297 .0015632 55.55 0.000 .083766 .0898935

wcharlsum_0 | .0969528 .0168772 5.74 0.000 .0638742 .1300315

AMI_0 | .0742306 .0216581 3.43 0.001 .0317816 .1166796

CHF_0 | .224861 .0199916 11.25 0.000 .1856781 .2640439

PVD_0 | .1944063 .0206416 9.42 0.000 .1539495 .2348631

CEVD_0 | .0631719 .0202286 3.12 0.002 .0235247 .1028192

dementia_0 | .528312 .0199737 26.45 0.000 .4891643 .5674598

COPD_0 | .1036814 .019019 5.45 0.000 .0664047 .140958

rheum_0 | -.1289828 .0237666 -5.43 0.000 -.1755645 -.0824011

PUD_0 | .0141996 .0252397 0.56 0.574 -.0352692 .0636685

LD_mild_0 | .562966 .0270554 20.81 0.000 .5099384 .6159935

LD_severe_0 | .8811587 .0578036 15.24 0.000 .7678658 .9944517

diab_0 | -.1567521 .0190852 -8.21 0.000 -.1941583 -.1193458

diab_compl_0 | .0515014 .023466 2.19 0.028 .0055089 .097494

RD_0 | .2168684 .0368935 5.88 0.000 .1445584 .2891784

cancer_0 | .8065647 .0343616 23.47 0.000 .7392172 .8739121

metastatic_0 | .6469169 .1018122 6.35 0.000 .4473687 .8464651

single | .734306 .005209 140.97 0.000 .7240965 .7445155

migrant | -.4264422 .0121552 -35.08 0.000 -.4502659 -.4026186

income_q |

2 | -1.088986 .0063092 -172.60 0.000 -1.101351 -1.07662

3 | -1.294179 .0067705 -191.15 0.000 -1.307449 -1.280909

4 | -1.350682 .0073309 -184.25 0.000 -1.36505 -1.336313

5 | -1.509252 .008371 -180.30 0.000 -1.525659 -1.492845

female | -.4568366 .0046849 -97.51 0.000 -.4660189 -.4476544

age_band |

2 | .2775345 .0142059 19.54 0.000 .2496915 .3053774

3 | .475774 .0132973 35.78 0.000 .4497117 .5018363

4 | .4475715 .0130353 34.34 0.000 .4220228 .4731202

5 | .6911666 .0128814 53.66 0.000 .6659194 .7164137

6 | 1.002263 .0126957 78.95 0.000 .9773796 1.027146

7 | 1.356909 .0126556 107.22 0.000 1.332105 1.381714

8 | 1.764771 .0129535 136.24 0.000 1.739382 1.790159

9 | 2.250177 .0137126 164.10 0.000 2.2233 2.277053

cost_pre1 | 3.58e-06 2.38e-07 15.05 0.000 3.11e-06 4.04e-06

cost_pre2 | 3.98e-06 3.57e-07 11.14 0.000 3.28e-06 4.68e-06

diag2 |

A1 | .7746134 .1522831 5.09 0.000 .4761439 1.073083

A2 | .0504122 .1996605 0.25 0.801 -.3409152 .4417396

A3 | 1.012212 .1890036 5.36 0.000 .6417723 1.382653

A4 | .5777804 .0345986 16.70 0.000 .5099684 .6455925

A6 | -.9817223 .2381333 -4.12 0.000 -1.448455 -.5149895

A7 | .2147993 .3358435 0.64 0.522 -.4434419 .8730404

A8 | -.064921 .1378567 -0.47 0.638 -.3351151 .2052731

B0 | -.1556971 .1059359 -1.47 0.142 -.3633276 .0519334

B1 | .1808107 .1415445 1.28 0.201 -.0966114 .4582329

B2 | .3613855 .1739167 2.08 0.038 .0205151 .7022559

B3 | .0457238 .0921389 0.50 0.620 -.1348651 .2263127

B4 | 1.530671 .2976862 5.14 0.000 .9472171 2.114125

B5 | .1862876 .3196899 0.58 0.560 -.4402932 .8128684

B9 | .4395144 .12548 3.50 0.000 .1935782 .6854505

C0 | .6232073 .0687354 9.07 0.000 .4884884 .7579262

C1 | 1.10896 .0359913 30.81 0.000 1.038418 1.179502

C2 | 1.467904 .0373472 39.30 0.000 1.394705 1.541103

C3 | 1.989303 .0357881 55.59 0.000 1.91916 2.059447

C4 | 1.171134 .054168 21.62 0.000 1.064967 1.277302

C5 | 1.004634 .0387498 25.93 0.000 .9286859 1.080582

C6 | .9471488 .0379359 24.97 0.000 .8727958 1.021502

C7 | 1.362446 .0363364 37.50 0.000 1.291228 1.433664

C8 | .583465 .049711 11.74 0.000 .4860333 .6808967

C9 | .973113 .0467941 20.80 0.000 .8813982 1.064828

D0 | -.1327448 .2552581 -0.52 0.603 -.6330414 .3675518

D1 | .1529304 .0734626 2.08 0.037 .0089463 .2969145

D2 | -.2512895 .1773858 -1.42 0.157 -.5989592 .0963802

D3 | .8378751 .0574583 14.58 0.000 .7252589 .9504913

D4 | 1.999486 .0481171 41.55 0.000 1.905178 2.093794

D5 | .5721922 .0449934 12.72 0.000 .4840068 .6603776

D6 | .8592392 .0357723 24.02 0.000 .7891268 .9293516

D7 | .8350978 .0851725 9.80 0.000 .6681628 1.002033

D8 | .6241263 .2078204 3.00 0.003 .2168057 1.031447

E0 | .1279929 .0792407 1.62 0.106 -.027316 .2833018

E1 | .4376036 .039688 11.03 0.000 .3598165 .5153908

E2 | .061314 .109216 0.56 0.575 -.1527455 .2753735

E3 | .8398006 .2313927 3.63 0.000 .3862792 1.293322

E4 | 1.457137 .1231798 11.83 0.000 1.215709 1.698565

E5 | .4089469 .1403896 2.91 0.004 .1337883 .6841054

E6 | .7485941 .0914797 8.18 0.000 .5692973 .927891

E7 | -.2833475 .2032395 -1.39 0.163 -.6816895 .1149945

E8 | .6947911 .0342115 20.31 0.000 .6277379 .7618444

F0 | .4791165 .0422006 11.35 0.000 .3964048 .5618282

F1 | .5556702 .0443032 12.54 0.000 .4688376 .6425028

F2 | .6970256 .120182 5.80 0.000 .4614733 .932578

F3 | .345215 .0825577 4.18 0.000 .1834048 .5070252

F4 | -.0869298 .1373299 -0.63 0.527 -.3560913 .1822318

F5 | .8429476 .3400196 2.48 0.013 .1765214 1.509374

F6 | 1.055736 .2752973 3.83 0.000 .5161634 1.595309

F9 | .4955469 .3075726 1.61 0.107 -.1072843 1.098378

G0 | .8436948 .0784064 10.76 0.000 .6900211 .9973685

G1 | 2.985182 .0632485 47.20 0.000 2.861218 3.109147

G2 | .9210073 .0614597 14.99 0.000 .8005486 1.041466

G3 | .7350881 .0712522 10.32 0.000 .5954364 .8747398

G4 | -.320053 .0397908 -8.04 0.000 -.3980415 -.2420646

G5 | -.0559137 .073092 -0.76 0.444 -.1991713 .0873439

G6 | .1312421 .1069938 1.23 0.220 -.0784619 .3409461

G7 | .5432566 .1519691 3.57 0.000 .2454026 .8411106

G8 | .5358235 .1155293 4.64 0.000 .3093903 .7622567

G9 | 1.043865 .0456337 22.87 0.000 .9544249 1.133306

H0 | -.4026064 .2799743 -1.44 0.150 -.9513459 .1461331

H1 | -.1725553 .1532952 -1.13 0.260 -.4730083 .1278977

H2 | -.126831 .1564438 -0.81 0.418 -.4334552 .1797932

H3 | -.8533279 .0924724 -9.23 0.000 -1.034571 -.6720852

H4 | -.2519265 .1036209 -2.43 0.015 -.4550197 -.0488333

H5 | -.256135 .1600166 -1.60 0.109 -.5697617 .0574917

H6 | -.0535206 .1752128 -0.31 0.760 -.3969313 .2898901

H7 | -.1928049 .5023456 -0.38 0.701 -1.177384 .7917743

H8 | -.6418436 .0769575 -8.34 0.000 -.7926775 -.4910097

H9 | -.3211527 .3827737 -0.84 0.401 -1.071375 .42907

I0 | .3716815 .1971584 1.89 0.059 -.0147418 .7581048

I1 | -.235239 .0426424 -5.52 0.000 -.3188165 -.1516615

I2 | .0578076 .034665 1.67 0.095 -.0101346 .1257498

I3 | .509618 .0423316 12.04 0.000 .4266496 .5925865

I4 | .141481 .0339502 4.17 0.000 .0749398 .2080222

I5 | .489706 .0371931 13.17 0.000 .4168089 .5626031

I6 | .4018828 .0348337 11.54 0.000 .33361 .4701556

I7 | .7919156 .0389295 20.34 0.000 .7156152 .8682159

I8 | .2094582 .04022 5.21 0.000 .1306285 .2882879

I9 | .3117942 .0697316 4.47 0.000 .1751228 .4484655

J0 | -.1940008 .085092 -2.28 0.023 -.3607781 -.0272235

J1 | .6943418 .0329827 21.05 0.000 .6296968 .7589868

J2 | .4267431 .0456764 9.34 0.000 .3372189 .5162672

J3 | -.4571409 .1128939 -4.05 0.000 -.678409 -.2358729

J4 | .5910159 .0359175 16.45 0.000 .5206189 .6614129

J6 | 1.616344 .0745348 21.69 0.000 1.470258 1.762429

J7 | .3040929 .2371268 1.28 0.200 -.160667 .7688529

J8 | 1.207649 .047737 25.30 0.000 1.114087 1.301212

J9 | 1.283286 .0347284 36.95 0.000 1.21522 1.351352

K0 | -.1981114 .2130325 -0.93 0.352 -.6156475 .2194246

K1 | .2234206 .108782 2.05 0.040 .0102119 .4366293

K2 | .41377 .0384287 10.77 0.000 .3384512 .4890888

K3 | -.3643663 .0533332 -6.83 0.000 -.4688975 -.2598352

K4 | -.1324793 .0496825 -2.67 0.008 -.2298551 -.0351034

K5 | .3423339 .0348917 9.81 0.000 .2739475 .4107203

K6 | .2097813 .0412495 5.09 0.000 .1289337 .2906288

K7 | .9443586 .0443674 21.28 0.000 .8574002 1.031317

K8 | .0557226 .03793 1.47 0.142 -.0186188 .1300639

K9 | .5837377 .04206 13.88 0.000 .5013016 .6661737

L0 | -.2834653 .0529336 -5.36 0.000 -.3872133 -.1797174

L1 | .4324333 .1616166 2.68 0.007 .1156706 .7491961

L2 | .3638297 .1456231 2.50 0.012 .0784137 .6492457

L3 | .0058122 .1604472 0.04 0.971 -.3086585 .3202829

L4 | -.2175159 .2452773 -0.89 0.375 -.6982507 .2632188

L5 | -.2749489 .1356292 -2.03 0.043 -.5407773 -.0091206

L6 | .9319076 .7106148 1.31 0.190 -.4608718 2.324687

L7 | -.1202046 .2922748 -0.41 0.681 -.6930527 .4526434

L8 | 1.182252 .0931341 12.69 0.000 .9997119 1.364791

L9 | .5652762 .0736064 7.68 0.000 .4210103 .709542

M0 | .089952 .0704717 1.28 0.202 -.0481699 .2280739

M1 | -.2156059 .0554052 -3.89 0.000 -.3241982 -.1070136

M2 | -.4600748 .1209514 -3.80 0.000 -.6971352 -.2230145

M3 | .033096 .064261 0.52 0.607 -.0928532 .1590452

M4 | .0300058 .0623665 0.48 0.630 -.0922302 .1522419

M5 | .029919 .0438034 0.68 0.495 -.0559341 .1157721

M6 | -.3211506 .0912343 -3.52 0.000 -.4999666 -.1423346

M7 | -.1622235 .0538098 -3.01 0.003 -.2676887 -.0567583

M8 | .4232055 .0515324 8.21 0.000 .3222038 .5242072

M9 | .6297277 .0993241 6.34 0.000 .4350561 .8243993

N0 | .5287333 .1067572 4.95 0.000 .3194931 .7379735

N1 | .5923966 .0386294 15.34 0.000 .5166844 .6681088

N2 | -.679098 .0700953 -9.69 0.000 -.8164823 -.5417138

N3 | .4482451 .0351607 12.75 0.000 .3793314 .5171588

N4 | -.1971104 .0646716 -3.05 0.002 -.3238643 -.0703565

N5 | -.5063087 .3582274 -1.41 0.158 -1.208422 .195804

N6 | -.6522621 .2324798 -2.81 0.005 -1.107914 -.19661

N7 | -.6240187 .167772 -3.72 0.000 -.9528458 -.2951916

N8 | -.7583752 .1158518 -6.55 0.000 -.9854406 -.5313098

N9 | .292447 .0931785 3.14 0.002 .1098204 .4750736

Q2 | .7819739 .1965056 3.98 0.000 .3968299 1.167118

Q4 | .6453573 .2540587 2.54 0.011 .1474114 1.143303

Q6 | .1895616 .2389627 0.79 0.428 -.2787968 .65792

Q8 | 1.20488 .4136021 2.91 0.004 .394235 2.015525

Q9 | 2.59903 .3640024 7.14 0.000 1.885599 3.312462

R0 | .3396212 .0364046 9.33 0.000 .2682695 .4109729

R1 | .674238 .034697 19.43 0.000 .6062331 .7422428

R2 | .2558646 .041209 6.21 0.000 .1750965 .3366327

R3 | .3935514 .0378335 10.40 0.000 .3193991 .4677036

R4 | -.0135889 .0421666 -0.32 0.747 -.096234 .0690561

R5 | .2664896 .0344928 7.73 0.000 .198885 .3340942

R6 | .9933065 .0508497 19.53 0.000 .893643 1.09297

R7 | .3522444 .0844661 4.17 0.000 .1866939 .5177948

R8 | 1.181402 .1556548 7.59 0.000 .8763242 1.48648

R9 | 2.415626 .0475417 50.81 0.000 2.322446 2.508806

S0 | .2047535 .0390081 5.25 0.000 .1282989 .281208

S1 | .3270188 .0801906 4.08 0.000 .1698481 .4841894

S2 | -.0124179 .0510842 -0.24 0.808 -.112541 .0877052

S3 | .1824181 .0423632 4.31 0.000 .0993878 .2654484

S4 | .0200283 .0426854 0.47 0.639 -.0636336 .1036901

S5 | -.5395429 .0436681 -12.36 0.000 -.6251308 -.4539549

S6 | -.7389855 .0737119 -10.03 0.000 -.8834581 -.5945129

S7 | .4371713 .0332099 13.16 0.000 .3720811 .5022615

S8 | -.344037 .042479 -8.10 0.000 -.4272944 -.2607796

S9 | -.3160283 .0861526 -3.67 0.000 -.4848842 -.1471724

T0 | -.0911383 .0727709 -1.25 0.210 -.2337666 .05149

T1 | .0468661 .0626228 0.75 0.454 -.0758722 .1696044

T2 | .2722956 .1222662 2.23 0.026 .0326583 .511933

T3 | .2949215 .078662 3.75 0.000 .1407469 .4490961

T4 | .4633732 .0556653 8.32 0.000 .3542712 .5724752

T5 | .2627067 .0866866 3.03 0.002 .0928042 .4326093

T6 | -.1176306 .0939269 -1.25 0.210 -.3017238 .0664627

T7 | .013047 .0604647 0.22 0.829 -.1054617 .1315557

T8 | -.0920595 .0389289 -2.36 0.018 -.1683587 -.0157603

T9 | .0553445 .1111669 0.50 0.619 -.1625386 .2732275

X6 | .1440136 .3567604 0.40 0.686 -.5552239 .8432512

Z0 | .5406396 .0325753 16.60 0.000 .4767931 .6044861

Z1 | -.8416354 1.001538 -0.84 0.401 -2.804614 1.121343

Z2 | .3256964 .585192 0.56 0.578 -.821259 1.472652

Z4 | -.2786016 .0793638 -3.51 0.000 -.4341518 -.1230514

Z5 | .2319603 .0363067 6.39 0.000 .1608005 .3031201

Z7 | .5318019 .0594144 8.95 0.000 .4153518 .6482519

Z8 | .740327 .0732452 10.11 0.000 .5967691 .8838849

Z9 | -.2450977 .0698155 -3.51 0.000 -.3819336 -.1082618

1.cohort2d2 | -.0197402 .0110167 -1.79 0.073 -.0413324 .0018521

1.cohort2d3 | -.0880956 .0110928 -7.94 0.000 -.1098371 -.066354

1.cohort2d4 | -.1340223 .0111804 -11.99 0.000 -.1559355 -.112109

1.cohort2d5 | -.2019186 .0112917 -17.88 0.000 -.2240499 -.1797873

periodvar | -.0384346 .000464 -82.83 0.000 -.0393441 -.0375252

cohort2d2#c.periodvar |

1 | -.0022373 .0006571 -3.40 0.001 -.0035252 -.0009494

cohort2d3#c.periodvar |

1 | -.0044496 .0006624 -6.72 0.000 -.0057479 -.0031513

cohort2d4#c.periodvar |

1 | -.0024875 .0006644 -3.74 0.000 -.0037897 -.0011853

cohort2d5#c.periodvar |

1 | -.0010026 .000666 -1.51 0.132 -.0023079 .0003028

_cons | -5.115438 .0348464 -146.80 0.000 -5.183736 -5.04714

---------------------------------------------------------------------------------------

**Part 2** estimates $\hat{\mu}_{1j}\left( X \right)$ in the person-periods in which the individual dies.

This is estimated using a two-part model with the first part consisting in a logit model (first output table below) for the probability of positive health care expenditure, and the second part consisting in a GLM model for positive HCE with gamma distribution and log link function (second output table below).

First part of **Part 2:**

Logistic regression Number of obs = 219,872

LR chi2(242) = 34048.40

Prob > chi2 = 0.0000

Log likelihood = -133137.41 Pseudo R2 = 0.1134

---------------------------------------------------------------------------------------

costperiod | Coef. Std. Err. z P>|z| [95% Conf. Interval]

----------------------+----------------------------------------------------------------

diagtot_0 | -.0026767 .0033837 -0.79 0.429 -.0093087 .0039553

wcharlsum_0 | -.0243922 .0380566 -0.64 0.522 -.0989817 .0501974

AMI_0 | .4167579 .0486863 8.56 0.000 .3213345 .5121813

CHF_0 | .1380911 .044597 3.10 0.002 .0506825 .2254997

PVD_0 | .2326403 .0460749 5.05 0.000 .1423351 .3229455

CEVD_0 | .0277791 .044655 0.62 0.534 -.059743 .1153012

dementia_0 | -.4946341 .0440056 -11.24 0.000 -.5808835 -.4083847

COPD_0 | .1382844 .0424874 3.25 0.001 .0550106 .2215581

rheum_0 | .0881181 .0524754 1.68 0.093 -.0147318 .190968

PUD_0 | .1537189 .0556811 2.76 0.006 .044586 .2628518

LD_mild_0 | .0739026 .0590798 1.25 0.211 -.0418918 .1896969

LD_severe_0 | .3636119 .129929 2.80 0.005 .1089559 .618268

diab_0 | .0056161 .042572 0.13 0.895 -.0778234 .0890556

diab_compl_0 | .0858541 .0519474 1.65 0.098 -.0159609 .187669

RD_0 | .2192927 .082572 2.66 0.008 .0574545 .381131

cancer_0 | -.0839224 .0772941 -1.09 0.278 -.2354162 .0675713

metastatic_0 | .0804669 .2293829 0.35 0.726 -.3691153 .5300491

single | -.1162143 .0106868 -10.87 0.000 -.13716 -.0952687

migrant | -.0650692 .0260047 -2.50 0.012 -.1160375 -.0141009

income_q |

2 | -.3223595 .0133863 -24.08 0.000 -.3485962 -.2961227

3 | -.3944293 .0143261 -27.53 0.000 -.4225079 -.3663508

4 | -.3634595 .0157262 -23.11 0.000 -.3942823 -.3326367

5 | -.3206687 .0181644 -17.65 0.000 -.3562703 -.285067

female | -.1242987 .0100811 -12.33 0.000 -.1440572 -.1045401

age_band |

2 | -.0658154 .031118 -2.12 0.034 -.1268056 -.0048252

3 | -.0700308 .0291199 -2.40 0.016 -.1271047 -.0129569

4 | -.1798127 .0284353 -6.32 0.000 -.2355449 -.1240805

5 | -.2450227 .0281067 -8.72 0.000 -.3001109 -.1899346

6 | -.2937196 .0277351 -10.59 0.000 -.3480794 -.2393598

7 | -.3710098 .0276608 -13.41 0.000 -.425224 -.3167956

8 | -.4969731 .0282277 -17.61 0.000 -.5522983 -.4416479

9 | -.6968399 .0297253 -23.44 0.000 -.7551005 -.6385793

cost_pre1 | 1.96e-06 5.13e-07 3.82 0.000 9.56e-07 2.97e-06

cost_pre2 | 1.41e-06 7.36e-07 1.91 0.056 -3.68e-08 2.85e-06

diag2 |

A1 | -.0267647 .3305796 -0.08 0.935 -.6746888 .6211595

A2 | .4035429 .4273737 0.94 0.345 -.4340941 1.24118

A3 | .2446046 .4032108 0.61 0.544 -.545674 1.034883

A4 | .4596014 .0741684 6.20 0.000 .3142341 .6049687

A6 | 1.611872 .6403106 2.52 0.012 .3568863 2.866858

A7 | -.1340537 .6842796 -0.20 0.845 -1.475217 1.20711

A8 | -.4051311 .2939152 -1.38 0.168 -.9811943 .1709321

B0 | .0513581 .2266173 0.23 0.821 -.3928036 .4955197

B1 | -.1248633 .302197 -0.41 0.679 -.7171585 .4674319

B2 | -.2248805 .3734086 -0.60 0.547 -.9567479 .506987

B3 | -.05929 .1985662 -0.30 0.765 -.4484726 .3298926

B4 | -.1379434 .6405468 -0.22 0.829 -1.393392 1.117505

B5 | 1.639604 1.078196 1.52 0.128 -.4736208 3.752829

B9 | -.2426048 .2643297 -0.92 0.359 -.7606814 .2754718

C0 | .0676692 .1443026 0.47 0.639 -.2151588 .3504971

C1 | .1198521 .0763427 1.57 0.116 -.0297769 .2694811

C2 | -.0141537 .0785266 -0.18 0.857 -.1680631 .1397557

C3 | .1655494 .0754636 2.19 0.028 .0176434 .3134554

C4 | .0337278 .1133157 0.30 0.766 -.188367 .2558225

C5 | .1542948 .0822047 1.88 0.061 -.0068234 .315413

C6 | -.0424021 .0800183 -0.53 0.596 -.1992351 .1144308

C7 | -.1667316 .0757909 -2.20 0.028 -.3152791 -.0181841

C8 | .1153653 .1057595 1.09 0.275 -.0919196 .3226502

C9 | .1889197 .0993483 1.90 0.057 -.0057995 .3836388

D0 | .8683257 .5892014 1.47 0.141 -.2864879 2.023139

D1 | .1753481 .1580116 1.11 0.267 -.1343489 .4850452

D2 | .2646513 .3795125 0.70 0.486 -.4791795 1.008482

D3 | -.2048496 .1209517 -1.69 0.090 -.4419105 .0322114

D4 | -.4519353 .0993153 -4.55 0.000 -.6465897 -.2572809

D5 | .0056935 .0956048 0.06 0.953 -.1816885 .1930755

D6 | -.002256 .0760609 -0.03 0.976 -.1513327 .1468207

D7 | .3167592 .1820802 1.74 0.082 -.0401114 .6736298

D8 | .0936337 .4811922 0.19 0.846 -.8494857 1.036753

E0 | .0318307 .1692787 0.19 0.851 -.2999495 .3636109

E1 | .0728417 .0848082 0.86 0.390 -.0933794 .2390628

E2 | -.013527 .2351792 -0.06 0.954 -.4744698 .4474158

E3 | .0395484 .5027547 0.08 0.937 -.9458326 1.024929

E4 | .5309259 .2751851 1.93 0.054 -.0084271 1.070279

E5 | -.2230575 .301908 -0.74 0.460 -.8147862 .3686712

E6 | -.069637 .1934059 -0.36 0.719 -.4487057 .3094316

E7 | -.2192458 .4389558 -0.50 0.617 -1.079583 .6410917

E8 | .022829 .0728684 0.31 0.754 -.1199906 .1656485

F0 | -.1046103 .0897223 -1.17 0.244 -.2804628 .0712421

F1 | -.1996364 .0946176 -2.11 0.035 -.3850835 -.0141894

F2 | -.1982762 .2530934 -0.78 0.433 -.6943302 .2977777

F3 | -.0712439 .1753365 -0.41 0.685 -.4148971 .2724093

F4 | -.0192143 .2906521 -0.07 0.947 -.5888819 .5504533

F5 | -.4283982 .6915174 -0.62 0.536 -1.783747 .926951

F6 | -.0862984 .5555594 -0.16 0.877 -1.175175 1.002578

F9 | -.9243049 .650394 -1.42 0.155 -2.199054 .3504438

G0 | .5050394 .1763071 2.86 0.004 .1594839 .8505949

G1 | .2092851 .1328378 1.58 0.115 -.0510721 .4696424

G2 | -.4079891 .131537 -3.10 0.002 -.6657969 -.1501814

G3 | -.2928223 .1519796 -1.93 0.054 -.590697 .0050523

G4 | -.0330305 .0843613 -0.39 0.695 -.1983756 .1323146

G5 | -.1824155 .1559085 -1.17 0.242 -.4879904 .1231595

G6 | -.072821 .2290406 -0.32 0.751 -.5217323 .3760903

G7 | .6035253 .3392318 1.78 0.075 -.0613568 1.268407

G8 | .2228033 .2493082 0.89 0.371 -.2658317 .7114384

G9 | -.0424242 .0960772 -0.44 0.659 -.230732 .1458835

H0 | -.4376365 .6153761 -0.71 0.477 -1.643751 .7684784

H1 | .5498588 .3300667 1.67 0.096 -.09706 1.196778

H2 | .4891883 .3397658 1.44 0.150 -.1767403 1.155117

H3 | .0311628 .1989729 0.16 0.876 -.3588168 .4211425

H4 | .3753457 .2256156 1.66 0.096 -.0668528 .8175441

H5 | .1711288 .3410621 0.50 0.616 -.4973406 .8395982

H6 | .0234077 .3686551 0.06 0.949 -.6991431 .7459584

H7 | 1.707065 1.233085 1.38 0.166 -.7097376 4.123867

H8 | .1413925 .1634498 0.87 0.387 -.1789631 .4617481

H9 | .7720631 .9348566 0.83 0.409 -1.060222 2.604348

I0 | .8308867 .4497687 1.85 0.065 -.0506438 1.712417

I1 | .2741908 .0916423 2.99 0.003 .0945751 .4538065

I2 | .3560635 .0745438 4.78 0.000 .2099604 .5021666

I3 | .444762 .0920343 4.83 0.000 .264378 .6251459

I4 | .3907495 .072708 5.37 0.000 .2482443 .5332547

I5 | .1678874 .0795466 2.11 0.035 .0119789 .3237959

I6 | .4137663 .0743585 5.56 0.000 .2680262 .5595064

I7 | .4517217 .084559 5.34 0.000 .2859891 .6174543

I8 | -.0714764 .0860196 -0.83 0.406 -.2400717 .0971189

I9 | .0694306 .1495043 0.46 0.642 -.2235926 .3624537

J0 | .0349767 .1816656 0.19 0.847 -.3210814 .3910347

J1 | .3213127 .0704903 4.56 0.000 .1831542 .4594711

J2 | .2566634 .0976276 2.63 0.009 .0653168 .44801

J3 | .3350012 .2460904 1.36 0.173 -.1473271 .8173294

J4 | .2632894 .0769469 3.42 0.001 .1124762 .4141026

J6 | .5467375 .159544 3.43 0.001 .234037 .859438

J7 | -.1539562 .5117586 -0.30 0.764 -1.156985 .8490721

J8 | .2997518 .1025596 2.92 0.003 .0987386 .500765

J9 | .5646959 .0745482 7.57 0.000 .4185842 .7108077

K0 | .6085571 .4871802 1.25 0.212 -.3462986 1.563413

K1 | .0823712 .2355643 0.35 0.727 -.3793264 .5440687

K2 | .1374764 .0824662 1.67 0.096 -.0241544 .2991072

K3 | .1213329 .115347 1.05 0.293 -.1047431 .347409

K4 | .3907211 .1070452 3.65 0.000 .1809163 .6005259

K5 | .2363415 .0746276 3.17 0.002 .0900741 .382609

K6 | .2074058 .0881411 2.35 0.019 .0346524 .3801591

K7 | .1380803 .0966871 1.43 0.153 -.051423 .3275835

K8 | .1944191 .0813551 2.39 0.017 .034966 .3538722

K9 | .2733795 .0898619 3.04 0.002 .0972535 .4495055

L0 | .0876497 .1134734 0.77 0.440 -.134754 .3100534

L1 | -.4641253 .3515772 -1.32 0.187 -1.153204 .2249534

L2 | .252834 .3145962 0.80 0.422 -.3637631 .8694311

L3 | -.0464885 .3388785 -0.14 0.891 -.7106782 .6177012

L4 | .8255638 .562315 1.47 0.142 -.2765534 1.927681

L5 | .0539417 .2870815 0.19 0.851 -.5087277 .616611

L7 | .5241455 .679113 0.77 0.440 -.8068916 1.855183

L8 | -.1805079 .1936389 -0.93 0.351 -.5600331 .1990173

L9 | -.1294463 .1563774 -0.83 0.408 -.4359404 .1770478

M0 | .0654455 .1507614 0.43 0.664 -.2300415 .3609324

M1 | -.1208365 .119482 -1.01 0.312 -.3550168 .1133439

M2 | -.2999302 .2574037 -1.17 0.244 -.8044321 .2045718

M3 | .1887801 .1386154 1.36 0.173 -.0829012 .4604613

M4 | .0301542 .1351351 0.22 0.823 -.2347058 .2950142

M5 | -.0645252 .0935632 -0.69 0.490 -.2479058 .1188553

M6 | -.0925834 .1948679 -0.48 0.635 -.4745175 .2893506

M7 | -.0483368 .1153849 -0.42 0.675 -.2744869 .1778134

M8 | -.0899329 .1100251 -0.82 0.414 -.3055781 .1257124

M9 | -.1477408 .2087501 -0.71 0.479 -.5568835 .261402

N0 | -.1834387 .2268048 -0.81 0.419 -.6279679 .2610906

N1 | .2271921 .0827964 2.74 0.006 .0649141 .3894701

N2 | .2392323 .1519495 1.57 0.115 -.0585833 .5370479

N3 | -.1973621 .0750002 -2.63 0.009 -.3443597 -.0503644

N4 | .0887551 .1377777 0.64 0.519 -.1812841 .3587944

N5 | -.1543969 .7823225 -0.20 0.844 -1.687721 1.378927

N6 | .132401 .4928869 0.27 0.788 -.8336395 1.098442

N7 | .5234247 .3638469 1.44 0.150 -.1897022 1.236552

N8 | .3223089 .2498304 1.29 0.197 -.1673496 .8119675

N9 | -.031066 .2012717 -0.15 0.877 -.4255513 .3634194

Q2 | .2599695 .4377485 0.59 0.553 -.5980018 1.117941

Q4 | -.4386652 .5295398 -0.83 0.407 -1.476544 .5992137

Q6 | .5077124 .5460879 0.93 0.353 -.5626003 1.578025

Q8 | -.0686366 .9154403 -0.07 0.940 -1.862867 1.725593

Q9 | -.8198063 .7511992 -1.09 0.275 -2.29213 .6525172

R0 | .4153793 .0783372 5.30 0.000 .2618412 .5689174

R1 | .119605 .0741991 1.61 0.107 -.0258226 .2650325

R2 | -.0616928 .0878427 -0.70 0.482 -.2338615 .1104758

R3 | .028903 .0805603 0.36 0.720 -.1289922 .1867983

R4 | -.0508574 .0899474 -0.57 0.572 -.2271511 .1254363

R5 | .0860088 .0737386 1.17 0.243 -.0585162 .2305338

R6 | .0786026 .1080116 0.73 0.467 -.1330962 .2903015

R7 | -.0031229 .1806973 -0.02 0.986 -.3572831 .3510373

R8 | .5795753 .3508991 1.65 0.099 -.1081743 1.267325

R9 | 1.086079 .1066645 10.18 0.000 .8770208 1.295138

S0 | .265932 .0833554 3.19 0.001 .1025584 .4293056

S1 | .357062 .1746421 2.04 0.041 .0147698 .6993543

S2 | .1229581 .1095863 1.12 0.262 -.0918271 .3377433

S3 | .036051 .0904641 0.40 0.690 -.1412553 .2133573

S4 | .0043167 .0911934 0.05 0.962 -.174419 .1830525

S5 | .0948936 .093253 1.02 0.309 -.087879 .2776662

S6 | .1560077 .1596773 0.98 0.329 -.156954 .4689693

S7 | -.0779675 .0708742 -1.10 0.271 -.2168783 .0609434

S8 | .0627883 .0908373 0.69 0.489 -.1152496 .2408262

S9 | .3022523 .186591 1.62 0.105 -.0634594 .667964

T0 | .1932722 .1567099 1.23 0.217 -.1138735 .5004179

T1 | .0820164 .1329548 0.62 0.537 -.1785702 .342603

T2 | .1705362 .262091 0.65 0.515 -.3431528 .6842252

T3 | -.0113571 .1684436 -0.07 0.946 -.3415005 .3187863

T4 | -.1079912 .1185005 -0.91 0.362 -.3402479 .1242655

T5 | -.297797 .1854235 -1.61 0.108 -.6612204 .0656263

T6 | .2498164 .2030185 1.23 0.219 -.1480926 .6477254

T7 | .0231552 .1281161 0.18 0.857 -.2279478 .2742581

T8 | .2061526 .0832715 2.48 0.013 .0429434 .3693618

T9 | -.1580489 .2388305 -0.66 0.508 -.6261482 .3100503

X6 | -.6015303 .7684267 -0.78 0.434 -2.107619 .9045584

Z0 | .0178098 .0696249 0.26 0.798 -.1186526 .1542721

Z2 | -1.326095 1.26372 -1.05 0.294 -3.802941 1.150751

Z4 | .1330445 .1705185 0.78 0.435 -.2011657 .4672547

Z5 | -.0911584 .0773276 -1.18 0.238 -.2427176 .0604009

Z7 | -.1532845 .1246376 -1.23 0.219 -.3975698 .0910008

Z8 | .2891092 .1601069 1.81 0.071 -.0246944 .6029129

Z9 | .1790761 .1508816 1.19 0.235 -.1166465 .4747986

1.cohort2d2 | .0586036 .0217649 2.69 0.007 .0159451 .1012621

1.cohort2d3 | .1594207 .0220118 7.24 0.000 .1162784 .202563

1.cohort2d4 | .0910076 .0221711 4.10 0.000 .0475531 .1344621

1.cohort2d5 | .0933235 .0224329 4.16 0.000 .0493557 .1372912

periodvar | -.0378934 .0008893 -42.61 0.000 -.0396365 -.0361503

cohort2d2#c.periodvar |

1 | .0009802 .0012247 0.80 0.423 -.0014201 .0033805

cohort2d3#c.periodvar |

1 | -.0042151 .0012322 -3.42 0.001 -.0066301 -.0018

cohort2d4#c.periodvar |

1 | -.0013971 .0012414 -1.13 0.260 -.0038301 .001036

cohort2d5#c.periodvar |

1 | -.0003644 .0012441 -0.29 0.770 -.0028027 .0020739

udiscrete |

2 | 2.071955 .0464375 44.62 0.000 1.980939 2.16297

3 | 1.960245 .0470497 41.66 0.000 1.868029 2.052461

4 | 1.983642 .0473467 41.90 0.000 1.890844 2.07644

5 | 2.076762 .0477043 43.53 0.000 1.983263 2.170261

6 | 2.171173 .047781 45.44 0.000 2.077524 2.264822

7 | 2.24853 .0477938 47.05 0.000 2.154856 2.342204

8 | 2.352207 .0481495 48.85 0.000 2.257835 2.446578

9 | 2.423473 .0480553 50.43 0.000 2.329286 2.517659

10 | 2.502203 .0481502 51.97 0.000 2.40783 2.596575

11 | 2.563216 .0483774 52.98 0.000 2.468398 2.658034

12 | 2.642554 .0483975 54.60 0.000 2.547697 2.737411

13 | 2.675783 .0484912 55.18 0.000 2.580742 2.770824

14 | 2.768882 .0488085 56.73 0.000 2.673219 2.864545

15 | 2.85085 .0488178 58.40 0.000 2.755169 2.946532

16 | 2.886111 .0488392 59.09 0.000 2.790388 2.981834

17 | 2.97772 .0490504 60.71 0.000 2.881582 3.073857

18 | 2.970386 .0490246 60.59 0.000 2.874299 3.066472

19 | 3.031397 .0493699 61.40 0.000 2.934634 3.12816

20 | 3.091532 .0495883 62.34 0.000 2.99434 3.188723

21 | 3.175305 .0494926 64.16 0.000 3.078301 3.272308

22 | 3.219707 .0496861 64.80 0.000 3.122324 3.31709

23 | 3.194635 .049772 64.19 0.000 3.097084 3.292187

24 | 3.301663 .0499441 66.11 0.000 3.203775 3.399552

25 | 3.32012 .0500894 66.28 0.000 3.221946 3.418293

26 | 3.336904 .0503262 66.31 0.000 3.238266 3.435541

27 | 3.430726 .0504499 68.00 0.000 3.331846 3.529607

28 | 3.404192 .050559 67.33 0.000 3.305098 3.503286

29 | 4.038515 .2083716 19.38 0.000 3.630114 4.446916

_cons | -1.373453 .0854106 -16.08 0.000 -1.540855 -1.206051

---------------------------------------------------------------------------------------

Second part of **Part 2:**

Generalized linear models Number of obs = 125,611

Optimization : ML Residual df = 125,367

Scale parameter = 1.533387

Deviance = 146016.0951 (1/df) Deviance = 1.164709

Pearson = 192236.1412 (1/df) Pearson = 1.533387

Variance function: V(u) = u^2 [Gamma]

Link function : g(u) = ln(u) [Log]

AIC = 20.14638

Log likelihood = -1265059.514 BIC = -1325911

---------------------------------------------------------------------------------------

| OIM

costperiod | Coef. Std. Err. z P>|z| [95% Conf. Interval]

----------------------+----------------------------------------------------------------

diagtot_0 | .0708703 .0025052 28.29 0.000 .0659602 .0757805

wcharlsum_0 | .0067901 .0293014 0.23 0.817 -.0506395 .0642197

AMI_0 | .2505231 .0356467 7.03 0.000 .1806569 .3203894

CHF_0 | -.040542 .0335143 -1.21 0.226 -.1062289 .0251449

PVD_0 | .0226136 .0344527 0.66 0.512 -.0449125 .0901397

CEVD_0 | .0287433 .0344043 0.84 0.403 -.0386879 .0961744

dementia_0 | -.3206983 .0350439 -9.15 0.000 -.389383 -.2520136

COPD_0 | .0150134 .0322713 0.47 0.642 -.0482371 .0782639

rheum_0 | .0308315 .0395271 0.78 0.435 -.0466402 .1083032

PUD_0 | .0958695 .0417824 2.29 0.022 .0139775 .1777615

LD_mild_0 | .0077319 .0425737 0.18 0.856 -.075711 .0911747

LD_severe_0 | .3022112 .0963579 3.14 0.002 .1133533 .4910692

diab_0 | -.0222491 .0325158 -0.68 0.494 -.085979 .0414808

diab_compl_0 | -.0216359 .0387184 -0.56 0.576 -.0975227 .0542508

RD_0 | .1003696 .0626287 1.60 0.109 -.0223804 .2231196

cancer_0 | -.2884953 .0593976 -4.86 0.000 -.4049124 -.1720781

metastatic_0 | -.3573761 .1764248 -2.03 0.043 -.7031624 -.0115897

single | -.0289429 .0078593 -3.68 0.000 -.0443469 -.0135389

migrant | .0075505 .0194397 0.39 0.698 -.0305505 .0456515

income_q |

2 | -.0813936 .0100326 -8.11 0.000 -.1010571 -.0617301

3 | -.1052763 .010987 -9.58 0.000 -.1268104 -.0837422

4 | -.0944599 .0119948 -7.88 0.000 -.1179693 -.0709505

5 | -.0625703 .0138155 -4.53 0.000 -.0896482 -.0354925

female | -.040003 .0075352 -5.31 0.000 -.0547717 -.0252342

age_band |

2 | -.0452531 .0222172 -2.04 0.042 -.0887979 -.0017083

3 | -.0630021 .0206789 -3.05 0.002 -.1035321 -.0224721

4 | -.1281111 .0202656 -6.32 0.000 -.1678309 -.0883912

5 | -.1663974 .0200851 -8.28 0.000 -.2057635 -.1270314

6 | -.2568396 .0198529 -12.94 0.000 -.2957505 -.2179287

7 | -.3838562 .0198743 -19.31 0.000 -.4228092 -.3449032

8 | -.5299097 .0204674 -25.89 0.000 -.570025 -.4897944

9 | -.6730523 .0220815 -30.48 0.000 -.7163313 -.6297734

cost_pre1 | -1.80e-06 3.56e-07 -5.08 0.000 -2.50e-06 -1.11e-06

cost_pre2 | -1.04e-06 5.04e-07 -2.06 0.039 -2.03e-06 -5.12e-08

diag2 |

A1 | -.3054075 .2492953 -1.23 0.221 -.7940173 .1832024

A2 | .4995299 .3148669 1.59 0.113 -.1175978 1.116658

A3 | .2327429 .2972948 0.78 0.434 -.3499442 .81543

A4 | .2650818 .0593499 4.47 0.000 .1487581 .3814056

A6 | -.1632227 .3480602 -0.47 0.639 -.8454081 .5189628

A7 | .0486118 .5568073 0.09 0.930 -1.04271 1.139934

A8 | .5885107 .2448856 2.40 0.016 .1085438 1.068478

B0 | .1555943 .1838766 0.85 0.397 -.2047973 .5159859

B1 | -.1009056 .2238705 -0.45 0.652 -.5396838 .3378725

B2 | .0863572 .3008838 0.29 0.774 -.5033642 .6760786

B3 | .0709728 .1601868 0.44 0.658 -.2429876 .3849332

B4 | .7788363 .4717187 1.65 0.099 -.1457154 1.703388

B5 | .9835818 .4169972 2.36 0.018 .1662824 1.800881

B9 | .074706 .222819 0.34 0.737 -.3620112 .5114231

C0 | .0177592 .1091553 0.16 0.871 -.1961811 .2316996

C1 | .0323348 .0611053 0.53 0.597 -.0874294 .1520991

C2 | -.1207535 .0627182 -1.93 0.054 -.2436788 .0021719

C3 | -.0805977 .0600265 -1.34 0.179 -.1982476 .0370521

C4 | -.2126467 .0870666 -2.44 0.015 -.383294 -.0419994

C5 | -.1722131 .0653031 -2.64 0.008 -.3002048 -.0442214

C6 | -.1162681 .0642948 -1.81 0.071 -.2422835 .0097473

C7 | -.0373793 .0612724 -0.61 0.542 -.1574711 .0827124

C8 | .1860206 .0815669 2.28 0.023 .0261525 .3458887

C9 | .4280759 .0768826 5.57 0.000 .2773888 .5787631

D0 | -.2995651 .3775867 -0.79 0.428 -1.039621 .4404912

D1 | .0231218 .1220093 0.19 0.850 -.216012 .2622555

D2 | -.7437515 .2895821 -2.57 0.010 -1.311322 -.176181

D3 | -.2034783 .0977639 -2.08 0.037 -.395092 -.0118646

D4 | -.1467649 .0828785 -1.77 0.077 -.3092038 .015674

D5 | .1213768 .0779289 1.56 0.119 -.031361 .2741145

D6 | -.0717103 .0615992 -1.16 0.244 -.1924426 .0490219

D7 | -.2771001 .1326015 -2.09 0.037 -.5369943 -.0172058

D8 | 1.068104 .3147154 3.39 0.001 .4512733 1.684935

E0 | -.2153858 .1380485 -1.56 0.119 -.485956 .0551843

E1 | .0702451 .0679264 1.03 0.301 -.0628883 .2033784

E2 | .0158563 .1872503 0.08 0.933 -.3511475 .3828601

E3 | .1432832 .3784308 0.38 0.705 -.5984275 .884994

E4 | -.1104558 .1775619 -0.62 0.534 -.4584708 .2375593

E5 | .1974205 .254062 0.78 0.437 -.3005318 .6953729

E6 | -.0346316 .1501088 -0.23 0.818 -.3288395 .2595763

E7 | -.5763768 .3619386 -1.59 0.111 -1.285763 .1330098

E8 | -.0761576 .0593346 -1.28 0.199 -.1924513 .0401361

F0 | .1149268 .0768725 1.50 0.135 -.0357406 .2655942

F1 | -.0440829 .0773358 -0.57 0.569 -.1956582 .1074924

F2 | .2048477 .2166635 0.95 0.344 -.219805 .6295004

F3 | -.0403517 .14396 -0.28 0.779 -.3225081 .2418048

F4 | -.5110037 .2329481 -2.19 0.028 -.9675736 -.0544338

F5 | -.622587 .6218242 -1.00 0.317 -1.84134 .5961661

F6 | -.0877454 .4715186 -0.19 0.852 -1.011905 .8364141

F9 | -.9379601 .6218605 -1.51 0.131 -2.156784 .2808641

G0 | .8099458 .1189422 6.81 0.000 .5768233 1.043068

G1 | -.2434754 .0962832 -2.53 0.011 -.4321869 -.0547638

G2 | -.2112588 .1145655 -1.84 0.065 -.4358031 .0132855

G3 | -.1345709 .1318756 -1.02 0.308 -.3930424 .1239006

G4 | .0528344 .069052 0.77 0.444 -.0825051 .1881738

G5 | .0107805 .1286197 0.08 0.933 -.2413094 .2628705

G6 | .4370814 .1822951 2.40 0.017 .0797896 .7943731

G7 | .4403676 .2293421 1.92 0.055 -.0091346 .8898699

G8 | .1515465 .1781744 0.85 0.395 -.1976689 .5007619

G9 | .2125517 .0758881 2.80 0.005 .0638138 .3612896

H0 | -.0203436 .5567998 -0.04 0.971 -1.111651 1.070964

H1 | .2622336 .249278 1.05 0.293 -.2263423 .7508095

H2 | -.0883751 .2539731 -0.35 0.728 -.5861532 .409403

H3 | .2051943 .1634218 1.26 0.209 -.1151065 .5254951

H4 | -.2451339 .1718588 -1.43 0.154 -.581971 .0917031

H5 | -.0020268 .2642231 -0.01 0.994 -.5198946 .515841

H6 | -.5045333 .2973651 -1.70 0.090 -1.087358 .0782917

H7 | -.9563914 .7172435 -1.33 0.182 -2.362163 .44938

H8 | .0103007 .1325056 0.08 0.938 -.2494055 .2700068

H9 | .309913 .5569086 0.56 0.578 -.7816077 1.401434

I0 | .798682 .3055624 2.61 0.009 .1997906 1.397573

I1 | .1789753 .0729088 2.45 0.014 .0360767 .321874

I2 | .0681853 .0593114 1.15 0.250 -.048063 .1844335

I3 | .2967913 .070018 4.24 0.000 .1595585 .434024

I4 | .2726545 .0586652 4.65 0.000 .1576728 .3876361

I5 | .0205523 .0630541 0.33 0.744 -.1030315 .1441361

I6 | .1903584 .0600113 3.17 0.002 .0727385 .3079784

I7 | .4124437 .0648864 6.36 0.000 .2852686 .5396187

I8 | -.0377297 .0697756 -0.54 0.589 -.1744873 .0990278

I9 | .1939798 .1178388 1.65 0.100 -.0369801 .4249397

J0 | -.0176047 .1461056 -0.12 0.904 -.3039665 .2687571

J1 | .1288877 .0571822 2.25 0.024 .0168127 .2409626

J2 | .179886 .0761525 2.36 0.018 .0306298 .3291422

J3 | .2073215 .1790657 1.16 0.247 -.1436409 .5582839

J4 | .1332035 .0613132 2.17 0.030 .0130319 .253375

J6 | .3117762 .1096935 2.84 0.004 .0967808 .5267715

J7 | -.168399 .3957885 -0.43 0.670 -.9441301 .6073322

J8 | .1859301 .0771698 2.41 0.016 .0346802 .33718

J9 | .3410425 .0590628 5.77 0.000 .2252816 .4568034

K0 | -.1534343 .3246278 -0.47 0.636 -.789693 .4828244

K1 | -.0861533 .1872849 -0.46 0.646 -.453225 .2809184

K2 | .1279682 .0658561 1.94 0.052 -.0011073 .2570438

K3 | .1308219 .0896133 1.46 0.144 -.044817 .3064607

K4 | .1111709 .0824065 1.35 0.177 -.0503429 .2726846

K5 | .191915 .0600634 3.20 0.001 .074193 .309637

K6 | .1137293 .0700188 1.62 0.104 -.023505 .2509636

K7 | .0389883 .071503 0.55 0.586 -.1011549 .1791316

K8 | .1755738 .0647464 2.71 0.007 .0486731 .3024744

K9 | -.059177 .0707039 -0.84 0.403 -.197754 .0794001

L0 | .1544922 .0909165 1.70 0.089 -.0237008 .3326852

L1 | .4824629 .3147914 1.53 0.125 -.1345169 1.099443

L2 | -.0701792 .2408835 -0.29 0.771 -.5423021 .4019438

L3 | .5922528 .2896229 2.04 0.041 .0246023 1.159903

L4 | -.4093252 .3619686 -1.13 0.258 -1.118771 .3001203

L5 | .3134723 .2293158 1.37 0.172 -.1359785 .762923

L6 | 1.70868 .8777141 1.95 0.052 -.0116076 3.428968

L7 | .4831346 .5567432 0.87 0.386 -.608062 1.574331

L8 | -.1110117 .1535178 -0.72 0.470 -.411901 .1898776

L9 | .0465267 .126619 0.37 0.713 -.2016421 .2946954

M0 | .1713427 .1199669 1.43 0.153 -.0637881 .4064736

M1 | .2617697 .1001109 2.61 0.009 .065556 .4579834

M2 | .143054 .2227258 0.64 0.521 -.2934806 .5795885

M3 | .309933 .1069955 2.90 0.004 .1002257 .5196404

M4 | .0425005 .1064259 0.40 0.690 -.1660904 .2510915

M5 | .0104972 .0762706 0.14 0.891 -.1389905 .159985

M6 | .0848336 .1623002 0.52 0.601 -.2332689 .4029361

M7 | .1383635 .0940435 1.47 0.141 -.0459584 .3226854

M8 | -.0444086 .0905103 -0.49 0.624 -.2218056 .1329883

M9 | .0494462 .1656658 0.30 0.765 -.2752528 .3741453

N0 | .181638 .1791347 1.01 0.311 -.1694596 .5327356

N1 | .0334554 .0649244 0.52 0.606 -.093794 .1607048

N2 | .195972 .1179304 1.66 0.097 -.0351673 .4271113

N3 | -.1109311 .0619019 -1.79 0.073 -.2322566 .0103943

N4 | .0594721 .1103791 0.54 0.590 -.1568668 .2758111

N5 | 1.022599 .6219906 1.64 0.100 -.1964798 2.241679

N6 | -.2381825 .3956508 -0.60 0.547 -1.013644 .5372788

N7 | -.006272 .264242 -0.02 0.981 -.5241768 .5116328

N8 | .0342571 .1969788 0.17 0.862 -.3518142 .4203284

N9 | .0162767 .1645786 0.10 0.921 -.3062914 .3388447

Q2 | .8287831 .3057856 2.71 0.007 .2294543 1.428112

Q4 | -.1693498 .4414614 -0.38 0.701 -1.034598 .6958987

Q6 | -.3039418 .3481266 -0.87 0.383 -.9862574 .3783738

Q8 | -.7969752 .7173234 -1.11 0.267 -2.202903 .6089528

Q9 | -1.503728 .7174589 -2.10 0.036 -2.909922 -.0975344

R0 | -.0057656 .0618814 -0.09 0.926 -.1270509 .1155197

R1 | -.1090875 .0597413 -1.83 0.068 -.2261784 .0080033

R2 | .0153613 .0722799 0.21 0.832 -.1263047 .1570274

R3 | -.0481558 .0654549 -0.74 0.462 -.176445 .0801333

R4 | -.0747709 .0739629 -1.01 0.312 -.2197354 .0701937

R5 | .0670728 .0598117 1.12 0.262 -.050156 .1843015

R6 | -.2783655 .0836879 -3.33 0.001 -.4423908 -.1143403

R7 | -.0157261 .1399803 -0.11 0.911 -.2900825 .2586302

R8 | -.2132932 .2259153 -0.94 0.345 -.656079 .2294926

R9 | -.2975031 .0713344 -4.17 0.000 -.4373159 -.1576903

S0 | .2042483 .0670196 3.05 0.002 .0728924 .3356042

S1 | .4978815 .1287927 3.87 0.000 .2454524 .7503105

S2 | .1632231 .0884098 1.85 0.065 -.0100569 .3365031

S3 | .11142 .0739576 1.51 0.132 -.0335342 .2563742

S4 | .0944283 .0751635 1.26 0.209 -.0528894 .241746

S5 | .0344865 .0773827 0.45 0.656 -.1171808 .1861539

S6 | -.1274131 .1268586 -1.00 0.315 -.3760513 .1212252

S7 | .1944079 .058138 3.34 0.001 .0804595 .3083563

S8 | .1569351 .0745316 2.11 0.035 .0108559 .3030144

S9 | .0008028 .144053 0.01 0.996 -.2815359 .2831416

T0 | .287244 .1217968 2.36 0.018 .0485267 .5259613

T1 | .0671469 .1086166 0.62 0.536 -.1457378 .2800315

T2 | .3470627 .2037373 1.70 0.088 -.052255 .7463804

T3 | -.0221538 .1347351 -0.16 0.869 -.2862297 .2419221

T4 | -.0442835 .0964042 -0.46 0.646 -.2332322 .1446653

T5 | .0428127 .1601736 0.27 0.789 -.2711217 .3567472

T6 | .2494983 .1543727 1.62 0.106 -.0530667 .5520633

T7 | .149554 .1046769 1.43 0.153 -.055609 .354717

T8 | .1538132 .0664547 2.31 0.021 .0235643 .2840621

T9 | -.0145257 .1991077 -0.07 0.942 -.4047695 .3757182

X6 | -.9655943 .6219609 -1.55 0.121 -2.184615 .2534266

Z0 | -.0772018 .05674 -1.36 0.174 -.1884102 .0340065

Z2 | -.4237183 1.240012 -0.34 0.733 -2.854098 2.006661

Z4 | .2278144 .1358904 1.68 0.094 -.0385259 .4941548

Z5 | .0754874 .0631574 1.20 0.232 -.0482989 .1992736

Z7 | -.1450848 .1036892 -1.40 0.162 -.348312 .0581424

Z8 | -.109693 .1140617 -0.96 0.336 -.3332499 .1138638

Z9 | .2011085 .1150223 1.75 0.080 -.024331 .426548

1.cohort2d2 | -.1351191 .0146065 -9.25 0.000 -.1637473 -.1064908

1.cohort2d3 | .0027277 .0147412 0.19 0.853 -.0261645 .0316199

1.cohort2d4 | .0756713 .0148449 5.10 0.000 .0465759 .1047667

1.cohort2d5 | .1504911 .015045 10.00 0.000 .1210033 .1799788

periodvar | -.0181227 .0006593 -27.49 0.000 -.0194149 -.0168304

cohort2d2#c.periodvar |

1 | .0084013 .0008983 9.35 0.000 .0066406 .010162

cohort2d3#c.periodvar |

1 | .0067384 .0009015 7.47 0.000 .0049714 .0085053

cohort2d4#c.periodvar |

1 | .0074836 .00091 8.22 0.000 .0057001 .0092672

cohort2d5#c.periodvar |

1 | .0018732 .0009137 2.05 0.040 .0000823 .003664

udiscrete |

2 | .2998184 .0514779 5.82 0.000 .1989235 .4007133

3 | .4440814 .0521168 8.52 0.000 .3419344 .5462285

4 | .5577415 .0523504 10.65 0.000 .4551366 .6603464

5 | .6398464 .0525034 12.19 0.000 .5369417 .7427512

6 | .6841856 .0524122 13.05 0.000 .5814595 .7869116

7 | .7210462 .0523298 13.78 0.000 .6184816 .8236108

8 | .7330029 .0524144 13.98 0.000 .6302725 .8357333

9 | .7556952 .0522745 14.46 0.000 .6532391 .8581514

10 | .7333862 .0522179 14.04 0.000 .631041 .8357314

11 | .755512 .0522218 14.47 0.000 .6531592 .8578649

12 | .7472109 .0521165 14.34 0.000 .6450645 .8493573

13 | .8119865 .0521566 15.57 0.000 .7097614 .9142117

14 | .8306235 .0521628 15.92 0.000 .7283862 .9328607

15 | .8143138 .0520415 15.65 0.000 .7123144 .9163132

16 | .8425295 .051992 16.20 0.000 .7406271 .944432

17 | .8391231 .0519455 16.15 0.000 .7373118 .9409345

18 | .843695 .0519641 16.24 0.000 .7418473 .9455427

19 | .8842645 .0519961 17.01 0.000 .782354 .9861751

20 | .8729284 .0519958 16.79 0.000 .7710185 .9748383

21 | .8981256 .051822 17.33 0.000 .7965562 .9996949

22 | .9347702 .0518309 18.03 0.000 .8331834 1.036357

23 | .9265177 .0519102 17.85 0.000 .8247755 1.02826

24 | .9402425 .0518292 18.14 0.000 .8386591 1.041826

25 | .9538992 .0518524 18.40 0.000 .8522703 1.055528

26 | .94506 .0519096 18.21 0.000 .843319 1.046801

27 | 1.001722 .0518065 19.34 0.000 .900183 1.103261

28 | 1.010022 .0518939 19.46 0.000 .908312 1.111733

29 | 1.211914 .1569585 7.72 0.000 .9042807 1.519547

_cons | 8.627799 .0769236 112.16 0.000 8.477032 8.778567

---------------------------------------------------------------------------------------

**Part 3** estimates $\hat{\mu}_{2j}\left( X \right)$ in the person-periods in which the individual survives.

This is estimated using a two-part model with the first part consisting in a logit model (first output table below) for the probability of positive HCE, and the second part consisting in a GLM model for positive HCE with gamma distribution and log link function (second output table below).

First part of **Part 3:**

Logistic regression Number of obs = 31,599,301

LR chi2(218) = 2102507.32

Prob > chi2 = 0.0000

Log likelihood = -13328214 Pseudo R2 = 0.0731

---------------------------------------------------------------------------------------

costperiod | Coef. Std. Err. z P>|z| [95% Conf. Interval]

----------------------+----------------------------------------------------------------

diagtot_0 | .0178685 .0004361 40.97 0.000 .0170137 .0187233

wcharlsum_0 | .1585856 .0035717 44.40 0.000 .1515851 .1655861

AMI_0 | -.1883249 .0046764 -40.27 0.000 -.1974905 -.1791593

CHF_0 | .000343 .0046932 0.07 0.942 -.0088556 .0095416

PVD_0 | .0495441 .0047577 10.41 0.000 .0402193 .058869

CEVD_0 | -.1697569 .0046294 -36.67 0.000 -.1788303 -.1606834

dementia_0 | -.3310493 .0058284 -56.80 0.000 -.3424727 -.319626

COPD_0 | .0398607 .0042407 9.40 0.000 .031549 .0481724

rheum_0 | .4535083 .0047878 94.72 0.000 .4441244 .4628923

PUD_0 | -.1210117 .0061496 -19.68 0.000 -.1330646 -.1089587

LD_mild_0 | .2368913 .0067312 35.19 0.000 .2236985 .2500841

LD_severe_0 | -.1155371 .0146803 -7.87 0.000 -.14431 -.0867642

diab_0 | .0791602 .0040784 19.41 0.000 .0711666 .0871538

diab_compl_0 | .3601304 .0049791 72.33 0.000 .3503717 .3698892

RD_0 | .3095835 .008257 37.49 0.000 .2934 .325767

cancer_0 | .2041015 .0073851 27.64 0.000 .189627 .2185761

metastatic_0 | -.5007639 .0219138 -22.85 0.000 -.5437141 -.4578136

single | -.0263088 .0011461 -22.96 0.000 -.0285551 -.0240626

migrant | -.0406814 .0022912 -17.76 0.000 -.0451721 -.0361906

income_q |

2 | -.0120177 .0016724 -7.19 0.000 -.0152955 -.0087399

3 | -.0138604 .0017365 -7.98 0.000 -.0172638 -.010457

4 | -.0198431 .001725 -11.50 0.000 -.0232241 -.0164621

5 | -.0852755 .0017666 -48.27 0.000 -.0887379 -.0818131

female | .0109923 .0010666 10.31 0.000 .0089019 .0130828

age_band |

2 | .0365367 .0019604 18.64 0.000 .0326945 .0403789

3 | .0608438 .0019156 31.76 0.000 .0570893 .0645984

4 | .0962249 .0019497 49.35 0.000 .0924034 .1000463

5 | .1220923 .0020252 60.29 0.000 .1181229 .1260617

6 | .1194407 .0021124 56.54 0.000 .1153005 .1235808

7 | .081289 .0022857 35.56 0.000 .0768092 .0857689

8 | .0164831 .0027348 6.03 0.000 .011123 .0218432

9 | -.0927969 .0038693 -23.98 0.000 -.1003805 -.0852132

cost_pre1 | .0000182 6.92e-08 263.17 0.000 .0000181 .0000183

cost_pre2 | .0000234 1.05e-07 221.51 0.000 .0000232 .0000236

diag2 |

A1 | .1017869 .0371936 2.74 0.006 .0288887 .1746851

A2 | -.0012659 .0309803 -0.04 0.967 -.0619861 .0594544

A3 | .0469311 .0537684 0.87 0.383 -.058453 .1523153

A4 | .0265396 .0065409 4.06 0.000 .0137197 .0393596

A6 | -.2030635 .0213536 -9.51 0.000 -.2449158 -.1612113

A7 | -.2470898 .0499324 -4.95 0.000 -.3449555 -.1492242

A8 | -.0903665 .0202507 -4.46 0.000 -.1300571 -.0506759

A9 | -.5395783 .0767064 -7.03 0.000 -.68992 -.3892365

B0 | .0101892 .0180308 0.57 0.572 -.0251504 .0455289

B1 | .0753479 .0243436 3.10 0.002 .0276354 .1230604

B2 | .3035233 .0273622 11.09 0.000 .2498944 .3571522

B3 | -.0427927 .0154718 -2.77 0.006 -.073117 -.0124685

B4 | .7937116 .0889933 8.92 0.000 .619288 .9681352

B5 | -.1437818 .0512628 -2.80 0.005 -.244255 -.0433085

B9 | .1588477 .026919 5.90 0.000 .1060874 .211608

C0 | .2051179 .0191225 10.73 0.000 .1676386 .2425972

C1 | .1360129 .0087974 15.46 0.000 .1187703 .1532556

C2 | .2288217 .0108066 21.17 0.000 .2076412 .2500022

C3 | .4505463 .0110048 40.94 0.000 .4289772 .4721154

C4 | .1592805 .0175578 9.07 0.000 .1248678 .1936931

C5 | .1673545 .0091307 18.33 0.000 .1494587 .1852503

C6 | .2618647 .0094213 27.80 0.000 .2433993 .28033

C7 | .2992661 .0106102 28.21 0.000 .2784705 .3200617

C8 | .1572078 .0120673 13.03 0.000 .1335563 .1808593

C9 | .42391 .0120555 35.16 0.000 .4002816 .4475383

D0 | .0929633 .0386667 2.40 0.016 .0171779 .1687486

D1 | .0924977 .0127236 7.27 0.000 .0675599 .1174355

D2 | -.1984659 .021519 -9.22 0.000 -.2406423 -.1562896

D3 | .3305612 .0125874 26.26 0.000 .3058903 .3552321

D4 | .6975439 .0147458 47.30 0.000 .6686427 .7264451

D5 | .1266669 .0100311 12.63 0.000 .1070064 .1463275

D6 | .2876753 .0076524 37.59 0.000 .2726769 .3026737

D7 | .4660364 .0205969 22.63 0.000 .4256672 .5064057

D8 | .4775491 .0334701 14.27 0.000 .4119489 .5431494

E0 | .0977148 .0135181 7.23 0.000 .0712198 .1242099

E1 | -.0297619 .0074218 -4.01 0.000 -.0443083 -.0152155

E2 | .4463935 .0165595 26.96 0.000 .4139375 .4788495

E3 | .3154427 .0598168 5.27 0.000 .1982039 .4326815

E4 | .0123021 .0517872 0.24 0.812 -.089199 .1138032

E5 | -.0536667 .0336464 -1.60 0.111 -.1196124 .012279

E6 | .0075091 .0262084 0.29 0.774 -.0438584 .0588766

E7 | -.0539696 .0268537 -2.01 0.044 -.1066019 -.0013373

E8 | .0128894 .0069652 1.85 0.064 -.0007622 .0265411

F0 | -.0111671 .0124615 -0.90 0.370 -.0355913 .013257

F1 | .0085421 .0084005 1.02 0.309 -.0079225 .0250067

F2 | -.0573961 .0330701 -1.74 0.083 -.1222122 .00742

F3 | -.0740484 .0180296 -4.11 0.000 -.1093858 -.0387111

F4 | -.0863772 .0214001 -4.04 0.000 -.1283207 -.0444337

F5 | .1166487 .0756307 1.54 0.123 -.0315848 .2648822

F6 | -.1156068 .0909536 -1.27 0.204 -.2938725 .0626589

F9 | .0475273 .0781491 0.61 0.543 -.1056422 .2006967

G0 | .1175008 .017177 6.84 0.000 .0838346 .1511671

G1 | .750049 .0281045 26.69 0.000 .6949651 .8051328

G2 | .4469077 .0142363 31.39 0.000 .4190051 .4748104

G3 | .2698404 .015294 17.64 0.000 .2398646 .2998161

G4 | -.0393558 .0069153 -5.69 0.000 -.0529096 -.025802

G5 | -.0418181 .011214 -3.73 0.000 -.0637971 -.0198392

G6 | .2077304 .0171258 12.13 0.000 .1741645 .2412963

G7 | .4028742 .0297343 13.55 0.000 .3445961 .4611524

G8 | .0060242 .0266488 0.23 0.821 -.0462064 .0582549

G9 | .2195355 .0110608 19.85 0.000 .1978566 .2412143

H0 | .3460471 .0307613 11.25 0.000 .2857562 .4063381

H1 | .0232473 .0244945 0.95 0.343 -.024761 .0712557

H2 | -.0227833 .0260976 -0.87 0.383 -.0739337 .0283671

H3 | -.0582328 .0095729 -6.08 0.000 -.0769953 -.0394703

H4 | .0383853 .0151712 2.53 0.011 .0086504 .0681202

H5 | .0481081 .0226366 2.13 0.034 .0037412 .092475

H6 | .0067528 .0249468 0.27 0.787 -.042142 .0556476

H7 | .0872942 .056387 1.55 0.122 -.0232223 .1978107

H8 | -.1173339 .0099392 -11.81 0.000 -.1368144 -.0978534

H9 | .0025072 .0531917 0.05 0.962 -.1017467 .1067611

I0 | .2234889 .0432249 5.17 0.000 .1387697 .3082082

I1 | -.0626671 .0071441 -8.77 0.000 -.0766692 -.048665

I2 | .0582438 .0061869 9.41 0.000 .0461176 .0703699

I3 | .1780802 .0084948 20.96 0.000 .1614307 .1947297

I4 | .1513246 .0060266 25.11 0.000 .1395126 .1631365

I5 | .0623254 .0079348 7.85 0.000 .0467735 .0778774

I6 | -.0518943 .0067246 -7.72 0.000 -.0650742 -.0387144

I7 | .0191384 .0083777 2.28 0.022 .0027184 .0355585

I8 | -.0316527 .0073822 -4.29 0.000 -.0461216 -.0171839

I9 | -.0126947 .0166497 -0.76 0.446 -.0453276 .0199381

J0 | -.0326225 .0120202 -2.71 0.007 -.0561817 -.0090634

J1 | .0513994 .0061081 8.41 0.000 .0394278 .0633711

J2 | .0633194 .0096657 6.55 0.000 .0443749 .0822638

J3 | -.1580176 .0141651 -11.16 0.000 -.1857806 -.1302546

J4 | .0303674 .0069668 4.36 0.000 .0167127 .0440221

J6 | .0571245 .0303965 1.88 0.060 -.0024516 .1167006

J7 | .2356538 .0615896 3.83 0.000 .1149404 .3563672

J8 | .1704162 .0125772 13.55 0.000 .1457654 .195067

J9 | .1598348 .0073784 21.66 0.000 .1453733 .1742962

K0 | -.0691846 .0305487 -2.26 0.024 -.1290589 -.0093103

K1 | -.1134242 .0191145 -5.93 0.000 -.150888 -.0759605

K2 | .0094785 .0076845 1.23 0.217 -.0055828 .0245398

K3 | -.1791145 .0077418 -23.14 0.000 -.1942882 -.1639408

K4 | -.1365285 .0086515 -15.78 0.000 -.1534852 -.1195718

K5 | .099585 .0063325 15.73 0.000 .0871735 .1119964

K6 | .027652 .007551 3.66 0.000 .0128522 .0424518

K7 | -.0568824 .0112721 -5.05 0.000 -.0789754 -.0347894

K8 | -.034319 .0065684 -5.22 0.000 -.0471928 -.0214453

K9 | .0381823 .0094628 4.03 0.000 .0196355 .0567291

L0 | -.115066 .0079146 -14.54 0.000 -.1305784 -.0995537

L1 | .2441649 .0381823 6.39 0.000 .1693291 .3190008

L2 | .1880317 .0278576 6.75 0.000 .1334318 .2426316

L3 | .0831189 .0287283 2.89 0.004 .0268124 .1394255

L4 | .2051236 .0307454 6.67 0.000 .1448638 .2653834

L5 | .0208616 .0170413 1.22 0.221 -.0125388 .0542619

L6 | -.2812709 .1643079 -1.71 0.087 -.6033085 .0407667

L7 | -.2127359 .0462816 -4.60 0.000 -.3034462 -.1220255

L8 | .2353025 .0284201 8.28 0.000 .1796001 .2910049

L9 | .0816208 .0164155 4.97 0.000 .049447 .1137946

M0 | .0434486 .0109989 3.95 0.000 .0218913 .065006

M1 | .0475968 .0089365 5.33 0.000 .0300816 .065112

M2 | .0381482 .0148109 2.58 0.010 .0091195 .067177

M3 | -.1532183 .0103786 -14.76 0.000 -.17356 -.1328765

M4 | .1951659 .0105405 18.52 0.000 .174507 .2158248

M5 | .0732095 .0073537 9.96 0.000 .0587966 .0876224

M6 | -.0407016 .0125964 -3.23 0.001 -.0653901 -.0160131

M7 | -.0148806 .0085045 -1.75 0.080 -.0315491 .0017879

M8 | .1077173 .0121515 8.86 0.000 .0839009 .1315337

M9 | .1733134 .0243442 7.12 0.000 .1255996 .2210272

N0 | .3617366 .0197623 18.30 0.000 .3230031 .40047

N1 | .1677565 .0076485 21.93 0.000 .1527658 .1827473

N2 | -.0533409 .0083463 -6.39 0.000 -.0696993 -.0369825

N3 | .0357474 .0068678 5.21 0.000 .0222867 .0492082

N4 | -.0215208 .0103549 -2.08 0.038 -.0418161 -.0012255

N5 | -.1352412 .0526325 -2.57 0.010 -.2383989 -.0320835

N6 | -.1407038 .0289384 -4.86 0.000 -.197422 -.0839856

N7 | -.1665523 .0167137 -9.97 0.000 -.1993106 -.133794

N8 | -.1948269 .013265 -14.69 0.000 -.2208258 -.168828

N9 | -.0357987 .0159517 -2.24 0.025 -.0670634 -.004534

Q1 | -.1877141 .1003372 -1.87 0.061 -.3843714 .0089433

Q2 | .3046158 .0372286 8.18 0.000 .2316492 .3775824

Q4 | -.1378054 .0537659 -2.56 0.010 -.2431846 -.0324262

Q6 | .4492693 .0334253 13.44 0.000 .3837569 .5147818

Q8 | .3486225 .0932438 3.74 0.000 .1658679 .531377

Q9 | -.6772264 .2358607 -2.87 0.004 -1.139505 -.2149479

R0 | .0360799 .0065954 5.47 0.000 .0231532 .0490066

R1 | .1060438 .0064154 16.53 0.000 .0934699 .1186177

R2 | .0120561 .0081276 1.48 0.138 -.0038737 .0279859

R3 | .1851782 .0073533 25.18 0.000 .1707659 .1995904

R4 | -.0357952 .0076842 -4.66 0.000 -.0508559 -.0207344

R5 | .0205869 .0062477 3.30 0.001 .0083416 .0328322

R6 | .1014138 .0133405 7.60 0.000 .0752669 .1275608

R7 | .1074669 .0169148 6.35 0.000 .0743145 .1406193

R8 | .3991158 .0485967 8.21 0.000 .303868 .4943636

R9 | .4748267 .0192645 24.65 0.000 .437069 .5125844

S0 | -.0896875 .0072743 -12.33 0.000 -.103945 -.07543

S1 | .0908051 .0150792 6.02 0.000 .0612504 .1203599

S2 | -.0730493 .0088412 -8.26 0.000 -.0903778 -.0557209

S3 | -.0122974 .0081609 -1.51 0.132 -.0282925 .0036977

S4 | -.0863354 .007603 -11.36 0.000 -.101237 -.0714338

S5 | -.1719916 .006742 -25.51 0.000 -.1852057 -.1587776

S6 | -.2349297 .0088575 -26.52 0.000 -.2522901 -.2175693

S7 | -.0384139 .0062543 -6.14 0.000 -.050672 -.0261558

S8 | -.1023011 .006648 -15.39 0.000 -.115331 -.0892713

S9 | -.1660027 .0119319 -13.91 0.000 -.1893888 -.1426166

T0 | -.0142687 .011493 -1.24 0.214 -.0367946 .0082571

T1 | -.1122254 .0111908 -10.03 0.000 -.134159 -.0902919

T2 | -.1655929 .0241189 -6.87 0.000 -.212865 -.1183207

T3 | -.0501324 .0137787 -3.64 0.000 -.0771382 -.0231266

T4 | -.0310093 .0114306 -2.71 0.007 -.0534129 -.0086058

T5 | -.1197208 .0162435 -7.37 0.000 -.1515575 -.087884

T6 | -.1423387 .0137818 -10.33 0.000 -.1693505 -.115327

T7 | -.0306361 .0102242 -3.00 0.003 -.0506752 -.0105971

T8 | .0516694 .0066824 7.73 0.000 .038572 .0647667

T9 | .0272635 .0177608 1.54 0.125 -.007547 .0620739

X6 | -.0987693 .0575156 -1.72 0.086 -.2114978 .0139591

Z0 | .0811911 .0058131 13.97 0.000 .0697975 .0925846

Z1 | -.2002575 .0870508 -2.30 0.021 -.370874 -.0296411

Z2 | .2396537 .1143366 2.10 0.036 .0155581 .4637494

Z4 | -.023499 .0119396 -1.97 0.049 -.0469002 -.0000978

Z5 | -.072273 .0071121 -10.16 0.000 -.0862126 -.0583335

Z7 | -.055741 .0148071 -3.76 0.000 -.0847623 -.0267197

Z8 | .0621983 .0189419 3.28 0.001 .0250729 .0993237

Z9 | -.0772335 .0110846 -6.97 0.000 -.0989589 -.0555082

1.cohort2d2 | .0159605 .0028316 5.64 0.000 .0104107 .0215102

1.cohort2d3 | .0063636 .0028017 2.27 0.023 .0008725 .0118548

1.cohort2d4 | .0244924 .002792 8.77 0.000 .0190201 .0299647

1.cohort2d5 | .0525269 .002776 18.92 0.000 .0470861 .0579677

periodvar | -.0524966 .0001103 -475.81 0.000 -.0527129 -.0522804

cohort2d2#c.periodvar |

1 | .0002881 .0001542 1.87 0.062 -.0000142 .0005903

cohort2d3#c.periodvar |

1 | .0041214 .0001509 27.31 0.000 .0038256 .0044172

cohort2d4#c.periodvar |

1 | .0044887 .0001499 29.93 0.000 .0041948 .0047825

cohort2d5#c.periodvar |

1 | .0050022 .0001486 33.67 0.000 .004711 .0052934

_cons | -1.070423 .0062976 -169.97 0.000 -1.082766 -1.05808

---------------------------------------------------------------------------------------

Second part of **Part 3:**

Generalized linear models Number of obs = 5355366

Optimization : ML Residual df = 5355147

Scale parameter = 4.046077

Deviance = 11167257.9 (1/df) Deviance = 2.085332

Pearson = 21667336.57 (1/df) Pearson = 4.046077

Variance function: V(u) = u^2 [Gamma]

Link function : g(u) = ln(u) [Log]

AIC = 18.42034

Log likelihood = -49323615.79 BIC = -7.18e+07

---------------------------------------------------------------------------------------

| OIM

costperiod | Coef. Std. Err. z P>|z| [95% Conf. Interval]

----------------------+----------------------------------------------------------------

diagtot_0 | .0739563 .0007546 98.01 0.000 .0724774 .0754353

wcharlsum_0 | .0444549 .0061487 7.23 0.000 .0324036 .0565062

AMI_0 | .1491388 .0081681 18.26 0.000 .1331297 .1651479

CHF_0 | .024376 .0080442 3.03 0.002 .0086096 .0401424

PVD_0 | .0033917 .0080948 0.42 0.675 -.0124739 .0192573

CEVD_0 | .03278 .007996 4.10 0.000 .0171081 .0484519

dementia_0 | -.1327507 .0103396 -12.84 0.000 -.153016 -.1124854

COPD_0 | -.0685752 .0072872 -9.41 0.000 -.0828578 -.0542926

rheum_0 | -.168981 .008002 -21.12 0.000 -.1846646 -.1532973

PUD_0 | .0199747 .0106846 1.87 0.062 -.0009667 .0409162

LD_mild_0 | .013835 .0114432 1.21 0.227 -.0085933 .0362633

LD_severe_0 | .2098265 .0249705 8.40 0.000 .1608852 .2587678

diab_0 | -.1764463 .0069907 -25.24 0.000 -.1901478 -.1627448

diab_compl_0 | -.1479275 .008378 -17.66 0.000 -.164348 -.1315071

RD_0 | .1876022 .0140102 13.39 0.000 .1601427 .2150616

cancer_0 | .0656193 .0126441 5.19 0.000 .0408373 .0904014

metastatic_0 | -.0660862 .0374325 -1.77 0.077 -.1394525 .0072801

single | .0783625 .0020146 38.90 0.000 .074414 .0823111

migrant | -.0559858 .0040605 -13.79 0.000 -.0639442 -.0480273

income_q |

2 | -.0507219 .0029198 -17.37 0.000 -.0564446 -.0449992

3 | -.0554907 .0030348 -18.28 0.000 -.0614389 -.0495426

4 | -.0666123 .003022 -22.04 0.000 -.0725353 -.0606894

5 | -.0642689 .0030947 -20.77 0.000 -.0703344 -.0582034

female | -.1392232 .001883 -73.94 0.000 -.1429139 -.1355325

age_band |

2 | .0689666 .003516 19.62 0.000 .0620754 .0758579

3 | .1205709 .0034154 35.30 0.000 .1138769 .1272649

4 | .1358077 .0034528 39.33 0.000 .1290404 .142575

5 | .1417442 .0035704 39.70 0.000 .1347464 .1487419

6 | .124787 .0037184 33.56 0.000 .117499 .1320749

7 | .1125223 .0040342 27.89 0.000 .1046154 .1204293

8 | .1224989 .0048703 25.15 0.000 .1129532 .1320446

9 | .1660099 .0069961 23.73 0.000 .1522979 .1797219

cost_pre1 | 2.89e-07 9.84e-08 2.94 0.003 9.65e-08 4.82e-07

cost_pre2 | -2.45e-07 1.37e-07 -1.79 0.074 -5.13e-07 2.37e-08

diag2 |

A1 | .3040214 .0661429 4.60 0.000 .1743836 .4336591

A2 | -.0225384 .0551848 -0.41 0.683 -.1306986 .0856218

A3 | .653768 .0912518 7.16 0.000 .4749177 .8326183

A4 | .2121218 .0116021 18.28 0.000 .189382 .2348616

A6 | .1047965 .0392612 2.67 0.008 .0278459 .1817471

A7 | -.0503823 .0925532 -0.54 0.586 -.2317832 .1310186

A8 | .156376 .0370408 4.22 0.000 .0837773 .2289746

A9 | -.7167602 .1452945 -4.93 0.000 -1.001532 -.4319883

B0 | .1278615 .0319102 4.01 0.000 .0653187 .1904043

B1 | -.0519807 .04294 -1.21 0.226 -.1361416 .0321802

B2 | -.0342244 .0458008 -0.75 0.455 -.1239924 .0555436

B3 | -.026535 .0277075 -0.96 0.338 -.0808407 .0277708

B4 | .4217765 .1272852 3.31 0.001 .1723021 .6712509

B5 | .0778482 .0922108 0.84 0.399 -.1028816 .2585779

B9 | .1772337 .0459497 3.86 0.000 .0871739 .2672935

C0 | .1728473 .0296416 5.83 0.000 .1147508 .2309437

C1 | .6596509 .0146986 44.88 0.000 .6308422 .6884597

C2 | .6758716 .0172548 39.17 0.000 .6420529 .7096904

C3 | .5107893 .0168125 30.38 0.000 .4778374 .5437413

C4 | .4053305 .0280515 14.45 0.000 .3503506 .4603105

C5 | .5289935 .0150605 35.12 0.000 .4994754 .5585116

C6 | .3086451 .0155449 19.86 0.000 .2781777 .3391124

C7 | .7304574 .0161376 45.26 0.000 .6988283 .7620865

C8 | .7295028 .0196495 37.13 0.000 .6909904 .7680152

C9 | 1.09837 .0190829 57.56 0.000 1.060969 1.135772

D0 | .0402931 .0666784 0.60 0.546 -.0903942 .1709804

D1 | .1621845 .0225633 7.19 0.000 .1179613 .2064078

D2 | -.1094134 .0396382 -2.76 0.006 -.187103 -.0317239

D3 | .5083365 .0217218 23.40 0.000 .4657625 .5509104

D4 | 1.066947 .0238902 44.66 0.000 1.020123 1.113771

D5 | .309926 .0177418 17.47 0.000 .2751528 .3446992

D6 | .3667978 .0132851 27.61 0.000 .3407594 .3928362

D7 | .4244227 .0328863 12.91 0.000 .3599669 .4888786

D8 | .2491547 .056741 4.39 0.000 .1379445 .360365

E0 | -.0432133 .0241459 -1.79 0.074 -.0905384 .0041117

E1 | .2442784 .0130496 18.72 0.000 .2187017 .2698552

E2 | -.0455036 .0281761 -1.61 0.106 -.1007277 .0097205

E3 | .5083238 .0922306 5.51 0.000 .3275552 .6890925

E4 | .2684682 .089993 2.98 0.003 .0920851 .4448512

E5 | .293965 .0604766 4.86 0.000 .1754331 .4124969

E6 | .2275785 .044491 5.12 0.000 .1403778 .3147793

E7 | .1420334 .048532 2.93 0.003 .0469124 .2371544

E8 | .182751 .0123232 14.83 0.000 .158598 .2069039

F0 | .2572802 .0225269 11.42 0.000 .2131283 .3014321

F1 | .1126436 .0151365 7.44 0.000 .0829766 .1423105

F2 | .2468181 .0602483 4.10 0.000 .1287336 .3649025

F3 | .0954432 .0325993 2.93 0.003 .0315498 .1593365

F4 | -.0502054 .0388393 -1.29 0.196 -.1263291 .0259182

F5 | .1016633 .1349172 0.75 0.451 -.1627696 .3660962

F6 | .2257572 .166957 1.35 0.176 -.1014724 .5529869

F9 | -.0882651 .1409966 -0.63 0.531 -.3646134 .1880831

G0 | .7291619 .03043 23.96 0.000 .6695202 .7888036

G1 | .7722063 .0444934 17.36 0.000 .6850009 .8594117

G2 | .1223785 .0244811 5.00 0.000 .0743965 .1703605

G3 | .4630424 .026581 17.42 0.000 .4109446 .5151402

G4 | -.1018006 .0122868 -8.29 0.000 -.1258823 -.0777189

G5 | .0380951 .0201407 1.89 0.059 -.00138 .0775702

G6 | .6687388 .029716 22.50 0.000 .6104965 .726981

G7 | .4723789 .0500111 9.45 0.000 .374359 .5703988

G8 | .4824982 .045233 10.67 0.000 .3938431 .5711532

G9 | .6098311 .0189626 32.16 0.000 .5726651 .646997

H0 | -.0440984 .0535327 -0.82 0.410 -.1490207 .0608238

H1 | -.0823393 .0436754 -1.89 0.059 -.1679415 .0032628

H2 | -.088913 .0471912 -1.88 0.060 -.1814062 .0035801

H3 | -.0545035 .017331 -3.14 0.002 -.0884715 -.0205354

H4 | -.117584 .0271463 -4.33 0.000 -.1707898 -.0643783

H5 | -.2673657 .0404684 -6.61 0.000 -.3466823 -.188049

H6 | .0260052 .04464 0.58 0.560 -.0614876 .113498

H7 | .1151359 .1014343 1.14 0.256 -.0836716 .3139435

H8 | -.1900814 .0180414 -10.54 0.000 -.225442 -.1547209

H9 | -.2321787 .0958747 -2.42 0.015 -.4200897 -.0442677

I0 | .4307386 .0728475 5.91 0.000 .2879602 .573517

I1 | -.0439727 .0128315 -3.43 0.001 -.0691221 -.0188234

I2 | .1779574 .0110477 16.11 0.000 .1563042 .1996106

I3 | .4882754 .0148733 32.83 0.000 .4591244 .5174265

I4 | .1081555 .0107319 10.08 0.000 .0871214 .1291895

I5 | .2521575 .0139194 18.12 0.000 .2248759 .279439

I6 | .374331 .011969 31.28 0.000 .3508722 .3977897

I7 | .5700777 .0146808 38.83 0.000 .5413039 .5988515

I8 | .0901028 .0131993 6.83 0.000 .0642326 .115973

I9 | .03129 .0295068 1.06 0.289 -.0265422 .0891223

J0 | .0529794 .0214663 2.47 0.014 .0109061 .0950526

J1 | .2168631 .010862 19.97 0.000 .195574 .2381522

J2 | .1919583 .0170957 11.23 0.000 .1584512 .2254653

J3 | -.0079191 .0257897 -0.31 0.759 -.058466 .0426278

J4 | .2375034 .0123218 19.28 0.000 .2133533 .2616536

J6 | .489715 .0540041 9.07 0.000 .3838689 .595561

J7 | .1109532 .091826 1.21 0.227 -.0690225 .2909288

J8 | .3939194 .0218065 18.06 0.000 .3511795 .4366593

J9 | .5037285 .012903 39.04 0.000 .4784391 .5290179

K0 | -.0076534 .0551772 -0.14 0.890 -.1157987 .1004918

K1 | .0527706 .0343299 1.54 0.124 -.0145148 .120056

K2 | .1159006 .0136603 8.48 0.000 .0891269 .1426744

K3 | -.0196173 .0140022 -1.40 0.161 -.047061 .0078265

K4 | .0346168 .0155966 2.22 0.026 .004048 .0651857

K5 | .1677662 .011246 14.92 0.000 .1457244 .1898079

K6 | .1356178 .0134143 10.11 0.000 .1093261 .1619094

K7 | .3723218 .0197052 18.89 0.000 .3337003 .4109433

K8 | .1372765 .0117553 11.68 0.000 .1142365 .1603165

K9 | .1833392 .0167229 10.96 0.000 .150563 .2161154

L0 | .0548736 .0142062 3.86 0.000 .0270299 .0827173

L1 | .2808759 .0656918 4.28 0.000 .1521223 .4096294

L2 | .345107 .0486084 7.10 0.000 .2498363 .4403776

L3 | .2887571 .0510634 5.65 0.000 .1886747 .3888395

L4 | .5678878 .0531378 10.69 0.000 .4637397 .6720359

L5 | .0034483 .0305459 0.11 0.910 -.0564205 .063317

L6 | .2699107 .3072466 0.88 0.380 -.3322817 .872103

L7 | .2181995 .0840012 2.60 0.009 .0535603 .3828387

L8 | .4799055 .0493815 9.72 0.000 .3831196 .5766915

L9 | .4438601 .028005 15.85 0.000 .3889713 .4987488

M0 | .3385047 .019082 17.74 0.000 .3011047 .3759047

M1 | .1187891 .0158744 7.48 0.000 .0876758 .1499024

M2 | -.1517723 .0265063 -5.73 0.000 -.2037238 -.0998208

M3 | .2540878 .0180213 14.10 0.000 .2187667 .2894089

M4 | .2964461 .018446 16.07 0.000 .2602925 .3325996

M5 | .1375828 .0131208 10.49 0.000 .1118665 .1632991

M6 | -.0588557 .0227151 -2.59 0.010 -.1033765 -.0143348

M7 | -.03679 .0152542 -2.41 0.016 -.0666877 -.0068922

M8 | .3047501 .0214239 14.22 0.000 .26276 .3467401

M9 | .4632545 .0418124 11.08 0.000 .3813037 .5452054

N0 | .4931018 .0332227 14.84 0.000 .4279865 .5582172

N1 | .3775773 .0133359 28.31 0.000 .3514394 .4037152

N2 | -.0467336 .0150457 -3.11 0.002 -.0762227 -.0172445

N3 | .0682832 .0122022 5.60 0.000 .0443674 .0921991

N4 | -.1067404 .0186245 -5.73 0.000 -.1432436 -.0702371

N5 | -.0097097 .0955815 -0.10 0.919 -.1970461 .1776266

N6 | .018662 .0505157 0.37 0.712 -.080347 .117671

N7 | -.0860474 .0306788 -2.80 0.005 -.1461769 -.025918

N8 | -.1645594 .0241487 -6.81 0.000 -.2118901 -.1172288

N9 | -.0236162 .0285828 -0.83 0.409 -.0796375 .0324051

Q1 | .4620324 .186435 2.48 0.013 .0966266 .8274382

Q2 | .7824124 .0649642 12.04 0.000 .655085 .9097399

Q4 | .2465291 .0974137 2.53 0.011 .0556017 .4374566

Q6 | .403947 .0551112 7.33 0.000 .295931 .5119629

Q8 | .3515141 .1580448 2.22 0.026 .041752 .6612761

Q9 | .1337317 .4503842 0.30 0.767 -.7490052 1.016469

R0 | -.0252391 .0117609 -2.15 0.032 -.04829 -.0021882

R1 | .1396001 .0113978 12.25 0.000 .1172609 .1619394

R2 | .0921768 .0145439 6.34 0.000 .0636712 .1206824

R3 | .0064792 .0129915 0.50 0.618 -.0189836 .031942

R4 | -.0833519 .0137727 -6.05 0.000 -.1103458 -.0563579

R5 | .0463737 .0111219 4.17 0.000 .024575 .0681723

R6 | .2072381 .0233788 8.86 0.000 .1614164 .2530598

R7 | .0740126 .0294094 2.52 0.012 .0163713 .1316539

R8 | .3865336 .0815543 4.74 0.000 .22669 .5463771

R9 | .622744 .0322142 19.33 0.000 .5596054 .6858827

S0 | .2150923 .013117 16.40 0.000 .1893835 .2408011

S1 | .5028893 .0270946 18.56 0.000 .4497848 .5559938

S2 | .0859434 .0159739 5.38 0.000 .0546351 .1172517

S3 | .1857481 .0146485 12.68 0.000 .1570375 .2144587

S4 | .0945398 .0136908 6.91 0.000 .0677064 .1213733

S5 | -.0502464 .0121373 -4.14 0.000 -.074035 -.0264578

S6 | -.1580487 .0161906 -9.76 0.000 -.1897816 -.1263158

S7 | .3460406 .0111787 30.96 0.000 .3241307 .3679505

S8 | .0699796 .0119445 5.86 0.000 .0465688 .0933904

S9 | .0260642 .021778 1.20 0.231 -.0166199 .0687483

T0 | .0992591 .0207889 4.77 0.000 .0585136 .1400046

T1 | .0365965 .0203128 1.80 0.072 -.0032158 .0764087

T2 | .2396055 .0444254 5.39 0.000 .1525333 .3266777

T3 | -.0214738 .0248764 -0.86 0.388 -.0702308 .0272831

T4 | -.0708395 .0205581 -3.45 0.001 -.1111326 -.0305464

T5 | -.0333217 .0296533 -1.12 0.261 -.0914411 .0247976

T6 | -.0715391 .025163 -2.84 0.004 -.1208576 -.0222205

T7 | -.104521 .0183649 -5.69 0.000 -.1405156 -.0685265

T8 | .2226508 .0117374 18.97 0.000 .199646 .2456557

T9 | -.0066606 .0316732 -0.21 0.833 -.068739 .0554178

X6 | -.4646862 .104196 -4.46 0.000 -.6689066 -.2604658

Z0 | .1461106 .0103601 14.10 0.000 .1258052 .1664159

Z1 | -.647259 .1605143 -4.03 0.000 -.9618612 -.3326568

Z2 | .176738 .1888937 0.94 0.349 -.1934867 .5469628

Z4 | -.0447654 .021205 -2.11 0.035 -.0863265 -.0032043

Z5 | .4258386 .0126581 33.64 0.000 .4010292 .450648

Z7 | .0572767 .0265958 2.15 0.031 .0051498 .1094036

Z8 | .2548855 .0315405 8.08 0.000 .1930672 .3167038

Z9 | .1489198 .0188444 7.90 0.000 .1119855 .1858541

1.cohort2d2 | -.0334398 .0042713 -7.83 0.000 -.0418114 -.0250682

1.cohort2d3 | .0099379 .0042205 2.35 0.019 .0016659 .01821

1.cohort2d4 | .0239432 .0041962 5.71 0.000 .0157189 .0321676

1.cohort2d5 | .0438998 .0041583 10.56 0.000 .0357497 .0520499

periodvar | -.0215641 .0001604 -134.47 0.000 -.0218784 -.0212498

cohort2d2#c.periodvar |

1 | .0026032 .0002243 11.60 0.000 .0021635 .0030429

cohort2d3#c.periodvar |

1 | -.0003921 .0002194 -1.79 0.074 -.0008221 .0000379

cohort2d4#c.periodvar |

1 | -.001027 .0002176 -4.72 0.000 -.0014535 -.0006005

cohort2d5#c.periodvar |

1 | -.006256 .0002148 -29.12 0.000 -.006677 -.005835

_cons | 8.153553 .0110944 734.92 0.000 8.131808 8.175298

---------------------------------------------------------------------------------------
