# Supplementary material for: Age, morbidity, or something else? A residual approach using microdata to measure the impact of technological progress on health care expenditure
Source: Health Econ. 2022 Mar 31;31(6):1184–201. doi: 10.1002/hec.4500 (PMC9314678; doi:10.1002/hec.4500)
Supplement: Supplementary file 2 — Supplementary Material 2 [file HEC-31-1184-s001.docx]

**Online Appendix 2**

**Table A1**. Residual Increment of annual HCE in a subsample of individuals with an initial health shock with at least two-day length of stay in the hospital. Exponentiated coefficients from GLM regression.

|  | Model 1 | Model 2 | Model 3 | Model 4 | Model 5 |  |
| --- | --- | --- | --- | --- | --- | --- |
|  |  |  |  |  |  |  |
| shock 2005-6 | baseline | baseline | baseline | baseline | baseline |  |
|  |  |  |  |  |  |  |
| shock 2007-8 | 1.0022 | 1.0011 | 0.9907** | 0.9853*** | 0.9855*** |  |
|  | (0.0050) | (0.0048) | (0.0043) | (0.0042) | (0.0042) |  |
| shock 2009-10 | 1.0974*** | 1.0912*** | 1.0590*** | 1.0486*** | 1.0488*** |  |
|  | (0.0057) | (0.0055) | (0.0049) | (0.0047) | (0.0047) |  |
| shock 2011-12 | 1.1676*** | 1.1581*** | 1.1079*** | 1.0925*** | 1.0926*** |  |
|  | (0.0061) | (0.0059) | (0.0052) | (0.0050) | (0.0050) |  |
| shock 2013-14 | 1.2396*** | 1.2279*** | 1.1584*** | 1.1311*** | 1.1310*** |  |
|  | (0.0066) | (0.0064) | (0.0056) | (0.0053) | (0.0053) |  |
| female |  | 0.8782*** | 0.9230*** | 0.9219*** | 0.9227*** |  |
|  |  | (0.0029) | (0.0029) | (0.0029) | (0.0030) |  |
| age |  | 1.1099*** | 1.0744*** | 1.0727*** | 1.0730*** |  |
|  |  | (0.0019) | (0.0017) | (0.0017) | (0.0017) |  |
| age sq. |  | 0.9992*** | 0.9994*** | 0.9995*** | 0.9995*** |  |
|  |  | (0.0000) | (0.0000) | (0.0000) | (0.0000) |  |
| total diagnoses |  |  | 1.1207*** | 1.1120*** | 1.1120*** |  |
|  |  |  | (0.0013) | (0.0013) | (0.0013) |  |
| Charlson index |  |  | 1.2170*** | 1.1654*** | 1.1654*** |  |
|  |  |  | (0.0166) | (0.0156) | (0.0156) |  |
| 15 comorbidities indicators | |  | yes | yes | yes |  |
|  |  |  |  |  |  |  |
| 175 primary diagnosis indicators | |  | yes | no | no |  |
|  |  |  |  |  |  |  |
| 1005 primary diagnosis indicators | |  | no | yes | yes |  |
|  |  |  |  |  |  |  |
| single |  |  |  |  | 1.0023 |  |
|  |  |  |  |  | (0.0032) |  |
| migrant |  |  |  |  | 0.9598*** |  |
|  |  |  |  |  | (0.0068) |  |
| income |  |  |  |  | 1.0002** |  |
|  |  |  |  |  | (0.0001) |  |
| income sq. |  |  |  |  | 1.0000** |  |
|  |  |  |  |  | (0.0000) |  |
| HCE one year before the shock | |  | 1.0000*** | 1.0000*** | 1.0000*** |  |
|  |  |  | (0.0000) | (0.0000) | (0.0000) |  |
| HCE two years before the shock | |  | 1.0000*** | 1.0000*** | 1.0000*** |  |
|  |  |  | (0.0000) | (0.0000) | (0.0000) |  |
|  |  |  |  |  |  |  |
| Constant | 16,435.7382*** | 602.0119*** | 738.0795*** | 2,038.3257*** | 2,073.5893*** |  |
|  | (56.8127) | (37.2955) | (44.4553) | (1,218.1927) | (1,255.4293) |  |
|  |  |  |  |  |  |  |
| Observations | 608,064 | 608,064 | 608,064 | 608,064 | 608,064 |  |
| BIC | -7491888 | -7504892 | -7617888 | -7630179 | -7630193 |  |
| Robust SE in parentheses | |  |  |  |  |  |
| *** p<0.01, ** p<0.05, * p<0.1 | |  |  |  |  |  |
